# A novel minimally humanized mouse model of aniridia for preclinical evaluation of CRISPR gene editing strategies

**DOI:** 10.3389/fgeed.2026.1910209

**Published:** 2026-09-18

**Authors:** Seyedeh Zeinab Mirjalili Mohanna, Andrea J. Korecki, Pardis Kazemian, Tess C. Lengyell, Bethany A. Adair, Blair R. Leavitt, Elizabeth M. Simpson

**Affiliations:** 1 Department of Medical Genetics, the University of British Columbia, Vancouver, BC, Canada; 2 Centre for Molecular Medicine and Therapeutics at BC Children’s Hospital, the University of British Columbia, Vancouver, BC, Canada; 3 The Djavad Mowafaghian Centre for Brain Health, the University of British Columbia, Vancouver, BC, Canada; 4 Division of Neurology, Department of Medicine, University of British Columbia Hospital, Vancouver, BC, Canada

**Keywords:** base editing (BE), cortical neurons, CRISPR genome editing, lipid nanoparticle (LNP), ocular disorder, paired box 6 gene, ribonucleoprotein complex (RNP)

## Abstract

**Introduction:**

Aniridia is a rare congenital vision-loss disorder primarily caused by variants in the paired box 6 (*PAX6*) gene. Current treatments do not maintain vision long term, nor address the underlying disease mechanism. CRISPR-mediated genome editing holds great promise for treating genetic diseases but requires demonstration of efficacy on human DNA to support translational relevance. The aim of this work was to generate and test a minimally humanized mouse model of aniridia, carrying a FLAG-tagged *Pax6* and the most common recurring pathogenic aniridia-patient variant, introduced for the first time in mice.

**Methods:**

Ocular assessments (gross examination, slit lamp imaging, and fluorescent and confocal microscopy) were performed in mice homozygous for humanization and heterozygous for the variant, paralleling patients. Mice were examined immediately post-wean and at adulthood, with the former chosen as a likely time for treatment initiation. As an exploratory proof-of-principle, a lipid nanoparticle-delivered adenine base editor (ABE8e) was tested to demonstrate the utility of the model.

**Results:**

Ocular assessments revealed the expected phenotypes for an aniridia mouse model, supporting successful generation and phenotypic validation of the humanized mouse. The ABE8e treatment resulted in a modest correction (4.8% ± 0.4% SEM) of the pathogenic variant in clinically relevant ex vivo cortical neurons.

**Discussion:**

Although designed for the development of genome editing therapies, this humanized mouse is also applicable to other therapies requiring interaction with human DNA or RNA. It also enables evaluation of nonsense-suppression drugs since the readthrough proteins retain the FLAG tag. More broadly, this methodology is applicable to other pathogenic variants for aniridia, CRISPRa approaches, and other genetic diseases for which CRISPR-based therapy holds promise.

## Introduction

Aniridia (OMIM:106210) is a rare congenital vision-loss disorder primarily caused by heterozygous loss-of-function variants and thus haploinsufficiency of the transcription factor paired box 6 (*PAX6*) (Jordan et al., 1992; Kit et al., 2021). PAX6 is a highly conserved transcription factor with a similar ocular expression pattern in mice and humans (Nishina et al., 1999; Collinson et al., 2003; Shaham et al., 2012). Postnatally, PAX6 expression within the eye is maintained in the retina (ganglion, amacrine, and horizontal cells), ciliary body, iris, lens, conjunctiva, and cornea (corneal epithelial cells) (Hickmott et al., 2016; Chanas et al., 2009). Aniridia is predominantly a panocular disorder, affecting multiple ocular structures including the cornea, iris, lens, retina, and optic nerve (Hall et al., 2025; Hingorani et al., 2012). It typically results in low vision and photophobia at birth due to retinal and iris abnormalities. Subsequently, vision-threatening glaucoma and aniridia-associated keratopathy (corneal disease) result in patients often becoming legally blind in their twenties to thirties. Current treatments focus on symptoms such as pharmacological approaches to glaucoma and dry eye, and surgical interventions for iris hypoplasia and keratopathy (Romano et al., 2025), but none maintain vision long term nor address the underlying disease mechanism. Critically, it has been shown in a mouse model of aniridia that increasing the levels of PAX6 protein, even postnatally, can at least partially correct the ocular phenotype (Gregory-Evans et al., 2013; Wang et al., 2017; Mirjalili Mohanna et al., 2020). Thus, there is a therapeutic window.

For aniridia, both gene augmentation and clustered regularly interspaced short palindromic repeats (CRISPR)-based therapeutic approaches are promising (Mirjalili Mohanna et al., 2022; Kaad et al., 2026; Djaksigulova et al., 2026). Gene augmentation to compensate for the overall reduction in gene expression would be variant independent and particularly applicable to a haploinsufficiency disorder such as aniridia (Kaad et al., 2026; Djaksigulova et al., 2026). Similarly, CRISPR activation (CRISPRa), in which a catalytically inactive Cas9 can be used to boost expression of the normal gene, is variant independent and dosage compensating (Riedmayr et al., 2022). However, both approaches may be limited by a narrow therapeutic index because of PAX6 dosage sensitivity (Manuel et al., 2007; Schedl et al., 1996). In contrast, CRISPR-mediated genome-editing therapies can correct pathogenic variants to the canonical sequence (Li et al., 2020), enabling gene expression under endogenous regulatory control in the correct cell type. However, the presence of over 600 different pathogenic variants in *PAX6* (Landrum et al., 2016; Lima Cunha et al., 2019), each requiring a variant-specific base-editing therapy, makes this approach daunting. On the positive side, just four of these variants account for 20% of aniridia cases (Lima Cunha et al., 2019). Furthermore, for another genetic disorder, a personalized gene-editing therapy developed for a specific variant in a single patient has already reached the clinic (Musunuru et al., 2025). The therapy was prepared in 6 months, delivered via LNP infusion, and the child is reported to be doing well. There are now plans to do it again for five more children, each with their own specific variant (Ledford, 2025). Both recombinant adeno-associated virus (rAAV) (Quinn et al., 2021; Pierce et al., 2024) and lipid nanoparticles (LNPs) have been used for *in vivo* delivery of CRISPR in clinical studies (Kotit, 2023; Suzuki et al., 2023).

Progress towards a long-term vision-saving therapy for aniridia is being made (Wang et al., 2017; Mirjalili Mohanna et al., 2022; Kaad et al., 2026; Djaksigulova et al., 2026; Abbasi et al., 2024). Essential for this progress has been the *Pax6*-variant mouse models of aniridia (Mirjalili Mohanna et al., 2020; Javidjam et al., 2024; Ton et al., 1992). These mice are important because typically one needs to demonstrate efficacy and safety in an animal model, before receiving approval for a clinical trial. One major challenge when undertaking therapy development with an animal model is the requirement to show efficacy on human DNA or RNA. This is particularly the case for genome-editing approaches such as CRISPR/Cas9, which require binding DNA (Ta et al., 2020). Unfortunately, for many genes the current collection of humanized mouse models fails to support genome-editing therapy development (Devoy et al., 2011; Zhang et al., 2025; Morito and Watamura, 2025; Rappaport and Johnson, 2014). Typically, humanized mice carry a multicopy random insertion of the entire locus under study. This often results in unexpected phenotypes due to the site of insertion, overexpression, and human versus mouse protein and regulatory differences. In addition, the presence of multiple copies makes these mice unsuitable models for genome-based editing correction. More applicable to gene editing approaches is the collection of human genes targeted into the genome as a single copy, and even better, when located in the endogenous mouse gene. Still, protein and regulatory differences can result in unexpected phenotypes, and large gene-replacement methods remain challenging.

To answer this challenge, we have developed the ‘‘CRISPR Humanized Minimally Mouse Models’’ (CHuMMMs) strategy (Adair et al., 2023; Gibson et al., 2025). Critical for the CHuMMMs concept is the minimization of the humanization; just enough to create a “landing pad” for the guide RNA (gRNA) and/or donor DNA of the gene-editing therapy, or CRISPRa. This has several advantages. 1) It allows the use of CRISPR technology to generate the mice, which is relatively fast compared to gene-replacement methods needed for larger insertions. 2) It places the humanization and causal variant in the context of the endogenous locus, maintaining single copy and normality of gene expression. 3) A matched humanized control without the causal variant can be made in parallel. This control strain will reveal any unexpected phenotypic impact of humanization alone, so it may be accounted for during therapy development, and provides the matched humanized version of the allele for dominant disease modelling. Finally, although genome-editing therapies can be developed using human cell lines, they do not allow examination of the entire phenotype, *in vivo* delivery, or the fulfillment of many preclinical requirements. Using a CHuMMM will ensure that the therapy is designed to bind human DNA, utilizing human protospacer adjacent motifs (PAMs) as needed, and providing proof of efficacy for immediate translation to human cells and patients.

In this study, we chose LNPs for CRISPR delivery due to their greater flexibility compared to rAAVs, whose limited packaging capacity (∼4.9 kb) (Dong et al., 1996; Wu et al., 2010) restricts the use of large editors such as *Streptococcus pyogenes* Cas9 (SpCas9) (∼4.1 kb) or ABE8e (∼4.8 kb) (Richter et al., 2020). LNPs are less constrained in payload size (Finn et al., 2018), and can deliver CRISPR in both plasmid and ribonucleoprotein (RNP) forms (Walther et al., 2024; Padilla et al., 2025). RNPs offer additional advantages including the use of chemically modified gRNAs for improved on-target editing (Hendel et al., 2015; Chen et al., 2021), natural clearance to reduce off-target effects (Jang et al., 2021; Liang et al., 2015; Kim et al., 2014), and no risk of genomic integration (Kim et al., 2014). Moreover, the engineered nature of LNPs allows repeated treatments (Finn et al., 2018; Kenjo et al., 2021; Rothgangl et al., 2021), overcoming the single-dose limitation of viral vectors caused by neutralizing antibodies (Jiang et al., 2006; Riviere et al., 2006).

The aim of this work was to generate a CHuMMM for aniridia and derive *ex vivo* cells from this mouse, which were then used in an exploratory proof-of-principle evaluation of an LNP-delivered CRISPR gene-editing strategy to demonstrate the utility of the model. Thus, we designed the CHuMMM to carry a new allele: *Pax6* ^*FHe9-*^ (FLAG-tagged humanized *Pax6* exon 9, pathogenic patient variant) (officially *Pax6* ^em8(PAX6*R240X)Ems^ (MGI:7579265)). As is typical, humanization of the entire *PAX6* locus resulted in a phenotype (Schedl et al., 1996; Kleinjan et al., 2001); thus we chose to humanize a single exon “landing pad” for CRISPR. Previously, a humanized control *Pax6* exon 9, non-variant (*Pax6* ^*He9+*^) allele was generated and resulted in no phenotype when carried heterozygously or homozygously (Mirjalili Mohanna et al., 2020; Adair et al., 2023). Exon 9 was chosen because it is the location of c.718C>T, the most frequently reported recurrent pathogenic *PAX6* variant associated with aniridia (Kit et al., 2021; Fokkema et al., 2011; Guo et al., 2022; Tyner et al., 2017). This variant accounts for 48 of 696 reported pathogenic variant entries (∼6.9%) in the *PAX6* Mutation Database (Richardson et al., 2016). It creates the nonsense change p. R240X, which is the type of variant found in ∼70% of human aniridia. (Lagali et al., 2019; Moosajee et al., 1993). The recurrence of this variant supports its potential as a clinically relevant target for development of variant-specific genome-editing therapies. This variant had not previously been introduced into mice, but it encodes a premature stop codon in exon 9 (human c.718C>T, p. R240X; MGI:7579265), similar to the small-eye variant (*Pax6* ^*Sey*^) which introduces a premature stop codon in exon 8 (human c.580G>T, p. G194X; MGI:1856155). Thus, we anticipated that the mutant phenotype would mirror that of the *Pax6* ^*Sey*^ mouse, in which the field has extensive expertise (Wang et al., 2017; Mirjalili Mohanna et al., 2022; Javidjam et al., 2024; Javidjam et al., 2026; Dashti et al., 2025; Cole et al., 2024). This is consistent with the CHuMMM not being designed to generate new phenotypes, but to allow rapid progression to therapy testing on human DNA.

Here we made certain choices for these experiments. Epistasis between *Pax6* variants and genetic background can have a profound effect on the timing and severity of the phenotype in mouse models of aniridia (Hickmott et al., 2018). We chose to characterize the *Pax6* ^*FHe9-*^ allele on the genetically invariant B6129F1 defined-hybrid background. This background has few epistatic interactions, and the polymorphic nature of B6129F1 is genetically naturalistic and more human-like (Hickmott et al., 2018). However, we note that even when the mouse genetic background is invariant, there remains variability in the aniridia phenotype (Hickmott et al., 2018). We characterized the mice at postnatal day (P) 21–22 and adulthood. The P21–22 time point, after weaning but before reaching sexual maturity, was chosen as a likely time for initiation of treatment. We demonstrate the CHuMMMs usefulness by deriving *ex vivo* cortical neurons and delivering a CRISPR strategy with the novel Incisive Delivery System. Cortical neurons represent a clinically relevant cell type, as the retina comprises multiple neuronal layers (Masland, 2012), and these cells have been shown to express PAX6 (Mirjalili Mohanna et al., 2022). Moreover, heterozygous *Sey* mice do not exhibit a cortical cellular phenotype (Tyas et al., 2003), and cortical neurons derived from these animals are grossly comparable to those from wild-type mice (Mirjalili Mohanna et al., 2022). Thus, these cells are well suited for this initial DNA correction study, as they avoid the confounding effects associated with unhealthy cells. Incisive Delivery System is an LNP-based platform that allows for the delivery of pre-complexed active Cas9/gRNA RNPs into target cells. The CRISPR strategy chosen here is based on our previously published work (Mirjalili Mohanna et al., 2022; Adair et al., 2023). It employs the adenine base editor (ABE8e), which enables selective single-base conversions without requiring donor DNA or producing double-stranded breaks (Gaudelli et al., 2017; Pradhan and Anoop, 2025; Liu et al., 2023; Lahr et al., 2023). This results in low rates of indels and reduced off-target editing, which makes base editors particularly advantageous for clinical therapies.

## Materials and methods

### Design of CRISPR components and assays to develop humanized mouse cells

The basic CRISPR approach and genotyping assays for CHuMMMs development were as previously described (Adair et al., 2023). In brief, the online tool Benchling (https://benchling.com/crispr) was used to design two gRNAs (cgEMS9, cgEMS18) using the *Sp*Cas9 nuclease, creating two double-stranded breaks in *Pax6* surrounding exon 9, to exchange ∼300 bp including all of mouse exon 9 and flanking intronic sequence with all of human exon 9 and flanking intronic sequence including the conserved splice sites. However, this time the DNA template also contained the patient pathogenic variant (c.718C>T, p. R240X) (template oEMS6346). The gRNAs and the DNA template used are listed in Supplementary Table 1. Assays were developed to screen for cell lines and mice that contained the humanized region (Inside Human, primers oEMS6223 and oEMS6224; 5′ insertion, primers oEMS6220 and oEMS6244; and 3′ insertion, primers oEMS6345 and oEMS6221). The primers used are listed in Supplementary Table 2.

### Targeting, picking, and screening single cell clones to produce humanized mouse embryonic stem cell lines

Male mouse embryonic stem cells (ESCs) (mEMS6738 and mEMS6740) were derived as previously described (Yang et al., 2009), from B6129F1 mice, which are first-generation defined hybrids produced by crossing C57BL/6J dams (RRID:IMSR_JAX:000664) (for brevity called B6), to 129S1/SvImJ sires (RRID:IMSR_JAX:002448) (for brevity called 129). These mice carried heterozygously the previously developed control allele *Pax6* ^*em3Ems*^ (MGI: 6423490), which for brevity and clarity will be called *Pax6* ^*Fax*^ (Mirjalili Mohanna et al., 2020; Mirjalili Mohanna et al., 2022). This allele carries a 3xFLAG tag at the ATG of the genomic *Pax6 locus*, without any additional variants, and was bred into the cells from both parental strains. We have previously demonstrated that mice carrying a heterozygous or homozygous *Pax6* ^*Fax*^ allele are phenotypically indistinguishable from wild-type animals, indicating that the tag does not measurably affect *Pax6* function (Mirjalili Mohanna et al., 2020). The allele was provided on the B6 strain background to the non-profit distributor Mutant Mouse Resource and Research Centres (MMRRC, Davis, CA, United States) as (RRID:MMRRC_066967-MU).

CRISPR components were delivered to ESCs using the Neon transfection system (Thermo Fisher, Waltham, MA, United States) as previously described (Mirjalili Mohanna et al., 2020; Adair et al., 2023). In brief, RNP complexes were formed by combining *Sp*Cas9 (PNA Bio, Thousand Oaks, CA, United States) with gRNAs (cgEMS9, cgEMS18) (Synthego), separately, and incubating at room temperature (RT) for 15 min. The DNA template (oEMS6346) was ordered as a single-stranded-oligodeoxynucleotide (ssODN) (Integrated DNA Technologies, Coralville, IA, United States) and added to the RNP complexes. 3 × 10^5^ ESCs were grown on mouse embryonic fibroblasts (MEFs) and electroporated using the Neon setting 14 (1200 V, 20 ms, 2 pulses). Final concentrations of reagents were: gRNA each 1.2228 µM, *Sp*Cas9 0.614 µM, DNA template 100 nM.

ESCs were plated directly onto 6 cm dishes, and individual clones were picked and plated onto 96-well plates on MEFs, until confluent. Clones were then passaged onto two sister plates, one on MEFs for freezing and one on 0.1% gelatin (Cat# G7-500, Thermo Fisher) for PCR. Once ESCs were confluent, cells were either frozen using ESC-freeze media (Yang et al., 2009), or lysed in tissue homogenization buffer (THB) as previously described (Mirjalili Mohanna et al., 2020; Adair et al., 2023; Yang et al., 2009).

Individual ESC clones were screened using the Inside Human, 5′, and 3′ assays. Cell lines that passed screening were thawed from the 96 well freeze plate and expanded further on MEFs for freezing, and on 0.1% gelatin for further screening including sequencing. DNA samples were purified using the PureLink PCR Purification Kit (Cat# K310001, Thermo Fisher), and sent for Sanger sequencing to confirm no undesired changes occurred surrounding the humanization region.

### Microinjection and chimera breeding to produce the *Pax6^FHe9-^* mouse strain

The ESC clone that passed all screening steps (mEMS6759) was shipped to The Jackson Laboratory (JAX, Bar Harbor, ME, United States) for expansion and microinjection into blastocysts from B6 albino (RRID:IMSR_JAX:000058) mice. Microinjected blastocysts were implanted into pseudo pregnant female mice, and resulting pups were assessed for % chimerism using ESC-derived coat color markers (Aw-J/a (agouti), Aw-J/Aw-J (agouti), a/a (black)).

Male chimeras were shipped from JAX, and all further mouse work was conducted at the pathogen-free Transgenic Animal facility at the Centre for Molecular Medicine and Therapeutics (CMMT) at the University of British Columbia (UBC) (Vancouver, BC, Canada). All further mouse work described in this paper was performed following protocols approved by the UBC Animal Care Committee (protocol numbers A19–0204 and A21-0140) in accordance with guidelines determined by the Canadian Council on Animal Care.

Standard mouse breeding strategies involved trios, two females and one male in a cage. The resulting pups were characterized by genotyping and sequencing using the assays described above. One chimera produced pups, and passed the new allele on to offspring. The new allele is named *Pax6* ^em8(PAX6*R240X)Ems^ (MGI:7579265), which for brevity and clarity will be called *Pax6* ^*FHe9-*^. The FLAG and the patient variant are 11.9 kb apart in the genome and thus tightly linked. The resulting strains were initiated from a single female, and subsequently backcrossed using males to either B6 or 129 for at least two generations to reduce off-target events. To enable timely access for the research community, they were provided to MMRRC as B6-*Pax6* ^*FHe9-*^ (officially C57BL/6J-*Pax6* ^em8(PAX6*R240X)Ems^, RRID:MMRRC_071760-JAX) and 129-*Pax6* ^*FHe9-*^ (officially 129-*Pax6* ^em8(PAX6*R240X)Ems^, RRID:MMRRC_071761-JAX), where further backcrossing is currently ongoing. The number of mice used was determined by the need to obtain at least one germline pup capable of establishing the *Pax6* ^*FHe9-*^ strain. Statistical tests were not used. Mice were excluded only by genotype and the investigator was aware of the genotype.

### Breeding, weight, and visual inspection of experimental cohort 1 mice

The experimental cohort 1 mice were produced by breeding the 129-*Pax6* ^*FHe9-*^ mice to 129 mice carrying the previously developed control allele *Pax6* ^*em7(PAX6)Ems*^ (MGI:7330074), the strain is 129S1.B6J-*Pax6* ^*em7(PAX6)Ems*^ (RRID:MMRRC_071299-MU), which for brevity and clarity will be called 129-*Pax6* ^*He9+*^ (Adair et al., 2023). This allele carries the same humanization of *Pax6* exon 9 as *Pax6* ^*FHe9-*^, but no FLAG tag nor pathogenic variant. The resulting 129-*Pax6* ^*FHe9-/He9+*^ mice were further bred to the previously developed control strain C57BL/6J-*Pax6* ^*em7(PAX6)Ems*^ (RRID:MMRRC_071298-MU), which for brevity and clarity will be called B6-*Pax6* ^*He9+*^ (Adair et al., 2023). This produced the experimental animals on the B6129F1 defined hybrid background: B6129F1-*Pax6* ^*FHe9-/He9+*^ and B6129F1-*Pax6* ^*He9+/He9+*^. Due to incomplete backcrossing, the experimental animals were not invariant 50% B6 and 50% 129, but instead on average 68.75% B6 and 31.25% 129.

Mating cages were closely monitored to accurately track the date of birth of B6129F1 experimental pups. Mice were aged to 21–22 days at which time ear notch samples were taken for genotyping, weight was recorded, and eyes were visually inspected for size (normal, small, or closed) and approximate level of corneal cloudiness (0%–100%). Genotyping was done with the previously described (Adair et al., 2023) Inside Mouse assay (primers oEMS6421 and oEMS6422), and *Ava*I/*Dde*I restriction fragment length polymorphism (RFLP) assays (Supplementary Table 2).

### Harvest at post-natal days 21–22 (histology only)

Following recording of weight and visual eye inspection, a subset of 73 mice (37 B6129F1-*Pax6* ^*He9+/He9+*^ and 36 B6129F1-*Pax6* ^*FHe9-/He9+*^) (Supplementary Table 3) were sacrificed by cervical dislocation, and eyes enucleated and placed in 4% paraformaldehyde (PFA) (Cat# P6148, Sigma-Aldrich, St. Louis, MO, United States) for 2 h, followed by 10% sucrose (Cat# S8501; Sigma-Aldrich) overnight. During the harvest procedure, the eyes of one B6129F1-*Pax6* ^*FHe9-/He9+*^ mouse were damaged and excluded from further processing and analysis. Additionally, a *Pax6* ^*FHe9-/He9+*^ mouse with an enlarged eye, suggestive of glaucoma, was also excluded from the analysis. Eyes were embedded in Tissue-Tek O.C.T. compound (Cat# 4583, Sakura Finetek, Torrance, CA, United States), sectioned at 20 μm, and directly mounted onto slides (Cat# 22037246, Thermo Fisher). Then, a light microscope (Olympus, Tokyo, Japan) was used in the selection of tissue sections. Representative slides were selected for nuclear staining. Where possible, corneal slides with clearly defined and intact epithelium and stromal layers in the peripheral cornea were selected, and retinal slides containing sections with the optic nerve head protruding into the retina and intact retinal sub-layers were selected. As we did not expect to see any phenotype in the B6129F1-*Pax6* ^*He9+/He9+*^ control mice, we only included 32 eyes (from 16 mice) for the histological analysis, whereas all 68 sectioned B6129F1-*Pax6* ^*FHe9-/He9+*^ eyes were subjected to histological analysis.

Briefly, sections were rinsed in 0.1 M phosphate buffered saline (PBS) (1 × 5 min) and 0.1% Triton X-100 (Cat# T8787, Sigma-Aldrich) (PBS-T) (1 × 10 min), and incubated with Hoechst (1:1000, Millipore Sigma, Burlington, MA, United States) for 20 min in a humid chamber at RT while protected from light. Staining media was then removed, and slides were washed in 0.1 M phosphate buffer (PB) (2 × 10 min) with a final wash in 0.01 M PB (1 × 10 min). Coverslips with ProLong Gold Antifade mounting solution (Cat# P10144, Thermo Fisher) were placed on slides, and slides were stored at 4 °C overnight. Imaging was performed on a BX61 fluorescent microscope (Olympus) at ×10 magnification in the DAPI channel using tile scans. The resulting images were processed using ImageJ (http://rsbweb.nih.gov/ij/), and Illustrator (Adobe, Mountain View, CA, United States).

### Harvest at 12 weeks (slit lamp and histology)

Following recording of weight and visual eye inspection, a subset of nine mice (five B6129F1-*Pax6* ^*He9+/He9+*^ and four B6129F1-*Pax6* ^*FHe9-/He9+*^) (Supplementary Table 3) were used for a longitudinal study where slit lamp images were taken at three timepoints (3 weeks, 6 weeks, and 12 weeks), and eyes were finally harvested at 12 weeks.

Slit lamp imaging was performed as previously described (Adair et al., 2023). In brief, mice were anesthetized using isoflurane, eyes covered with 1% Isopto Tears ophthalmic solution (ALCON, Geneva, Switzerland), and a Micron IV Retinal Imaging Microscope (Phoenix Research Labs, Pleasanton, CA, United States) with the anterior segment slit lamp attachment was used to image the left eyes. All images were taken at the same setting. RAW images were processed with a custom preset in the Lightroom (Adobe).

Following the 12-week slit lamp imaging, mice were sacrificed by cervical dislocation, and eyes were immediately enucleated and directly fixed in 4% PFA for 2 h. The embedding and sectioning of the eyes were carried out as described above. Eye sections were rinsed in PBS (2 × 5 min) and PBS-T (1 × 10 min), and blocked in PBS-T + 0.3% bovine serum albumin (Cat# A2058, Sigma-Aldrich) + 10% goat serum (Cat# G9023, Sigma-Aldrich) at RT for 1 h. Subsequently, sections were incubated with rabbit anti-PAX6 (1:1000; Cat# 901301, BioLegend, San Diego, CA, United States) at 4 °C overnight. The following day, sections were washed with PBS-T (3 × 10 min) and incubated with goat anti-rabbit immunoglobulin G (IgG) (1:1000; Cat# A11037, Thermo Fisher) and Hoechst (1:1000) at RT for 2 h. Finally, sections were rinsed with 0.1 M PB (3 × 10 min), and once in 0.01 M PB buffer (1 × 10 min). Coverslips with ProLong Gold Antifade mounting solution were placed on eye sections, and slides were stored at 4 °C overnight. Immunofluorescence imaging of eye sections was carried out at ×20 magnification using a Leica TCS SP8 Super-Resolution Confocal Microscope (Leica Microsystems, Wetzlar, Germany). Z-stack images were taken at 0.5 μm intervals and subsequently deconvoluted using Huygens Professional Software (Scientific Volume Imaging, Hilversum, Netherlands). The resulting images were processed using ImageJ, and Illustrator (Adobe).

### Isolation and culture of *ex vivo* cortical neurons from experimental cohort 2 mice

The experimental cohort 2 mouse production paralleled that of cohort 1 but with timed pregnancies to be used for neuronal isolation on embryonic day 17.5. The genotypes of dams and studs used for the pair matings were B6-*Pax6* ^*He9+/He9+*^ and 129-*Pax6* ^*FHe9-/He9+*^, respectively. Cortices of pups were micro-dissected and pooled based on genotype (either *Pax6* ^*He9+/He9+*^ or *Pax6* ^*FHe9-/He9+*^) to generate primary neuron cultures. 24-well culture plates were pre-coated with 100 μg/mL poly-D-lysine (Cat# P6407, Sigma-Aldrich). Neurons were plated at a seeding density of 1.5 × 10^5^ cells/well in 1 mL of media. Cultures were maintained in a cell culture incubator at 37 °C with 5% CO_2_ and received a half-media change on day *ex vivo* (DEV) 3.

### Preparation of lipid nanoparticles

To make the LNPs, ethanolic solutions of the following lipids were prepared at a concentration of 10 mM: 1,2-dioleyloxy-3-dimethylaminopropane (DODMA), 1,2-dioleoyl-sn-glycero-3-phosphoethanolamine (DOPE), 1,2-distearoyl-sn-glycero-3-phosphocholine (DSPC) (lipids from Avanti Polar Lipids, Alabaster, AL, United States), and cholesterol (Cat# C3045, Sigma-Aldrich). DODMA, DOPE, DSPC, and cholesterol were mixed at a molar ratio of 20:30:10:40. The lipid solution was combined with an aqueous solution of 25 mM sodium acetate (pH 4) using NanoAssemblr Ignite (Cytiva, Vancouver, Canada) at a total flow rate of 20 mL/min with a 3:1 v/v ratio (aqueous:lipid mixture). The resulting mixture was dialyzed in 100× volume excess of 25 mM sodium acetate (pH 4) overnight at 4 °C. Following dialysis, empty LNPs were concentrated using an Amicon centrifugation filter (Cat# UFC801008, Sigma-Aldrich). The final lipid concentration was adjusted to 10 mM, as determined using a cholesterol assay (Cat# CA97000-018, WAKO, Richmond, VA, United States).

### CRISPR strategy for targeting the pathogenic patient variant

The ABE8e protein was generated through plasmid overexpression followed by purification, as previously described (Adair et al., 2023). The *PAX6*-targeting gRNA (cgEMS25) used in this study to correct the pathogenic patient variant (c.718C>T, p. R240X) was also previously designed and validated (Adair et al., 2023). Briefly, candidate gRNAs were designed using Benchling and selected based on predicted on-target efficiency, minimized off-target potential, and proximity to the target base to ensure placement of the patient variant within the optimal editing window of ABE8e. These gRNAs were then delivered into mouse ESCs homozygous for the variant by electroporation to confirm editing efficiency, and cgEMS25 was selected as the most effective guide for further use. In the present study, delivery was instead carried out in cortical neurons heterozygous for the variant, reflecting patient genotypes, using LNPs.

### Ribonucleoprotein complex formation and lipid nanoparticle encapsulation

RNP complex formation and LNP encapsulation were performed as previously described (Adair et al., 2023). In brief, to form RNP complexes at a final concentration of 50 nM, an equimolar ratio of chemically modified cgEMS25 (Synthego, Menlo Park, CA, United States) was mixed with purified ABE8e protein and incubated for 5 min at RT. For the preparation of 100 nM of either luciferase targeting (negative control) or *PAX6* targeting (experimental) RNP complexes, twice the volumes of ABE8e and gRNA were used while maintaining equimolar ratios. Transfection-competent vesicles (TCVs) were mixed with the RNPs at a 1:1.67 ABE8e:TCV ratio for the 50 nM, and a 1:0.83 ratio for the 100 nM preparations. The mixtures were incubated for a final 5 min. The sequences of the gRNAs used in this experiment are listed in Supplementary Table 1.

The size distribution and polydispersity index of LNPs encapsulating the luciferase targeting RNP strategy at 50 nM and 100 nM concentrations were measured using a Malvern Zetasizer Ultra instrument (Worcestershire, UK) with a He-Ne laser (λ = 632 nm) at a detection angle of 173° (Supplementary Table 4). While the LNP characterizations reflect the final formulated particles used in the experiments, an important limitation should be noted. The loaded particles were transported to an external facility for size distribution and polydispersity measurements, which may have promoted particle aggregation and contributed to the larger size ranges observed. To further investigate this possibility, LNPs were encapsulated with the same gRNA and RNP at the measurement facility and analyzed immediately, which yielded a smaller and more uniform size distribution (Supplementary Table 5). Although these particles were not used in the *ex vivo* gene editing experiment, they likely better reflect the size characteristics, which has also been previously reported and replicated (Adair et al., 2023). Notably, LNPs containing 100 nM RNP still exhibited increased particle size compared to the lower concentration. This observation suggests that higher RNP loading may influence particle stability or assembly, and highlights the need for future optimization of the LNP:RNP ratio when working with high RNP concentrations.

### *Ex vivo* genome editing

B6129F1-*Pax6* ^*He9+/He9+*^ and B6129F1-*Pax6* ^*FHe9-/He9+*^ *ex vivo* cortical neurons were transfected on DEV 6. Transfection materials were prepared immediately prior to the treatment. Complete neurobasal medium containing 2 μg/mL recombinant human ApoE4 (Cat# 350–04, Peprotech, Cranbury, NJ, United States) was added to the 50 nM and 100 nM *PAX6* targeting RNP, as well as 100 nM luciferase targeting RNP to achieve a final volume of 500 μL. Half (500 μL) of the neuronal conditioned medium from each well was collected and kept at 37 °C. The transfection mixtures were then added to the wells containing neurons, bringing the total volume to 1 mL per treatment. Neurons were returned to the incubator for 2 h. Following incubation, the entire medium was removed from each well and replaced with 500 μL of the saved neuronal conditioned medium. Each well was then supplemented with 500 μL of fresh complete neurobasal medium and returned to the incubator until they were collected on DEV 9. Untreated control cells underwent the same media changes as the treated conditions.

### DNA isolation, Sanger sequencing, and analysis pipeline for quantifying editing outcomes

At DEV 9, each well of the 24-well plate was gently rinsed twice with 1 mL PBS prior to the addition of 200 μL THB. Cells were thoroughly scraped using a 200 μL pipette tip and transferred to 1.5 mL Eppendorf tubes. Cell lysis was performed using THB as previously described (Yang et al., 2009).

The region surrounding the humanized exon 9 (265 bp) was PCR-amplified directly from whole cell lysates using primers oEMS6223 and oEMS6224 (Supplementary Table 2). PCR products were purified using the PureLink PCR Purification kit (Thermo Fisher) following the manufacturer’s protocol. Bidirectional Sanger sequencing was carried out at the CMMT DNA Sequencing Core facility, with the sequencing operator blinded to sample identity to avoid bias.

Chromatograms were analyzed using SnapGene software (Insightful Science, San Diego, CA, United States; http://snapgene.com). Base editing outcomes were quantified using EditR software (http://baseeditr.com/), which extracts peak height data from Sanger sequencing chromatograms corresponding to base conversions at the target site (Kluesner et al., 2018). Editing efficiency was calculated by normalizing peak height data to a luciferase-targeting negative control to determine the percentage of variant correction in experimental samples. Quantification was performed by an investigator blinded to treatment groups, and no samples were excluded from the analysis.

### Phenotyping and genotyping of mice from B6-*Fey* experimental crosses

A FLAG tag was previously added to the *Pax6* ^*Sey*^ creating the FLAG-tagged *Sey* (*Pax6* ^*Fey*^) allele, designed to enable the detection of the corrected protein after successful genome editing (Mirjalili Mohanna et al., 2020; Mirjalili Mohanna et al., 2022; Adair et al., 2023). B6-*Pax6* ^*Fey/+*^ mice were crossed with B6-*Pax6* ^*He9+/+*^ mice to produce pups with the following genotypes: *+*/*+*, *Fey*/*+*, *He9* ^*+/+*^, and *Fey*/*He9* ^*+*^. In a separate study, B6-*Pax6* ^*Fey/+*^ mice were crossed with B6-*Pax6* ^*Fax/+*^ mice to produce pups with the following genotypes: *+*/*+*, *Fey*/*+*, *Fax*/*+*, and *Fey*/*Fax*. Cages were checked regularly. In some litters, pups were found cannibalized by their parents between P1–3. These pups were therefore excluded from the study, as they could not be used for phenotyping. At P19–31, pups were ear-notched for identification, and visual inspection of the eyes was immediately performed to assess phenotypes. The investigator was blinded to the genotypes while scoring the phenotypes. Collected ear notches were subsequently used for genotyping, which was performed as previously described (Mirjalili Mohanna et al., 2020; Mirjalili Mohanna et al., 2022; Adair et al., 2023).

### Statistical analysis

GraphPad Prism version 10.4.0 for Windows (GraphPad Software, San Diego, CA, United States, http://www.graphpad.com) was used for the statistical analyses and generation of graphs. Statistical analyses were performed using two-tailed t-test, chi-square test, and two-tailed Fisher’s exact test, and P ≤ 0.05 was considered to be statistically significant.

## Results

### Generating a novel FLAG-tagged minimally humanized mouse model of aniridia: *Pax6^FHe9-^*

The FLAG-tagged allele was introduced here into the new mouse model via ESCs, which were derived from a *Pax6* ^*Fax*^ homozygous embryo (Mirjalili Mohanna et al., 2020; Mirjalili Mohanna et al., 2022). The humanized *Pax6* exon 9 allele was recreated in the ESCs, using the same methodology as previously described (Figure 1A), (Adair et al., 2023) but now the humanization region contained the pathogenic patient variant (c.718C>T, p. R240X). In brief, a dual-gRNA strategy was used, and homology-directed repair was accomplished by electroporation with CRISPR RNPs and a ssODN template. A 512 bp ssODN mediated the exchange of a 312 bp region of mouse DNA sequence with human sequence, via flanking 100 bp mouse-specific homology arms. This resulted in the FLAG tag and patient variant being 11.9 kb apart in the genome and thus tightly linked.

**FIGURE 1:**
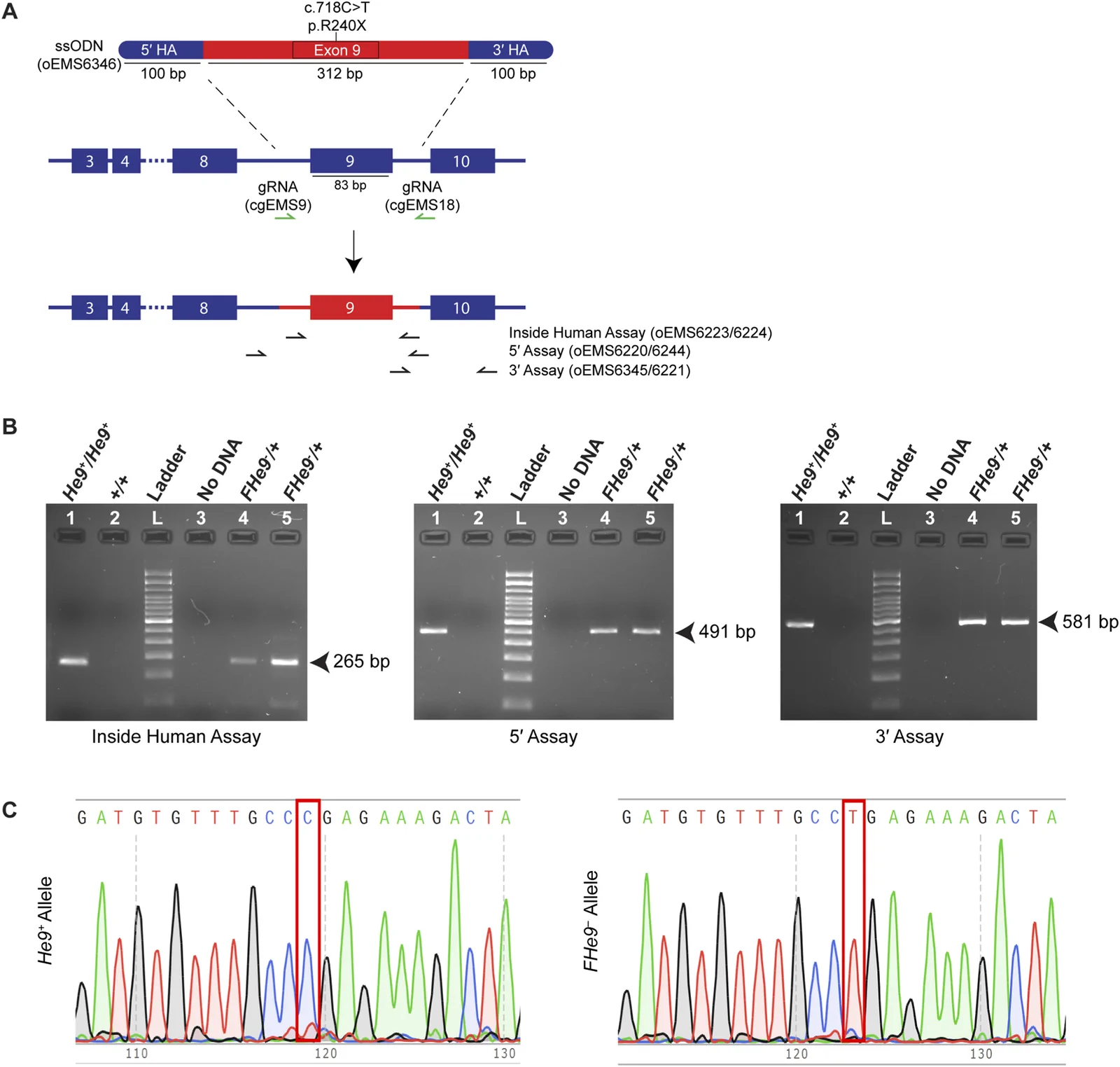
Generation of the new mouse model of aniridia. **(A)** Schematic of making the humanized *Pax6* exon 9 allele carrying a pathogenic patient variant (c.718C>T, p. R240X), not to scale. A CRISPR strategy with two gRNAs (cgEMS9 and cgEMS18) and a 512 bp ssODN was used to humanize a region spanning mouse *Pax6* exon 9. The ssODN (oEMS6346) consisted of 100-bp mouse 5′ and 3′ homology arms (HA) (blue) flanking a 312 bp human DNA sequence (red) containing the pathogenic patient variant. Horizontal black arrows show the forward and reverse primers used in three different PCR assays to confirm the successful humanization of *Pax6* exon 9. The reverse primer in the 5′ assay binds to the mouse–human junction. cgEMS#, CRISPR guide EMS#; gRNA, guide RNA; oEMS#, oligonucleotide EMS#; ssODN, single-stranded oligodeoxynucleotide donor. **(B)** Genotyping results of the first-pedigree mouse, which was used to “found” the colony. The expected band sizes are noted on the right-hand side of each gel. Lane 1: 129-*Pax6* ^*He9+/He9+*^ positive control, 2: B6-*Pax6* ^*+/+*^ negative control, L: 100 bp DNA ladder, 3: no DNA negative control, 4: first-pedigree mouse B6(B6129)-*Pax6* ^*FHe9−/+*^ (ID: 20822-2-1), 5: potential first-pedigree mouse B6(B6129)-*Pax6* ^*FHe9−/+*^. **(C)** Sanger sequencing confirms humanization and presence of pathogenic patient variant in the first-pedigree mouse. The human-specific “Inside Human Assay” from A was used to amplify DNA for sequencing from ear notches of the (left panel) 129-*Pax6* ^*He9+/He9+*^ positive control, and (right panel) first pedigree mouse B6(B6129)-*Pax6* ^*FHe9−/+*^. The red box shows the site of the patient variant.

A total of 224 single clones were picked from plates of electroporated cells (Supplementary Table 6). Of these, 187 clones were successfully expanded and frozen in 96 wells. PCR screening of the 187 clones with the inside-human assay confirmed insertion of the ssODN in 18 clones (Figure 1A). Of those 18 clones, 6 were positive for the 5′ and 3′ assays indicating the correct insertion (Figure 1A). These 6 clones were subsequently expanded for freezing (on MEFs) and Sanger sequencing (on gelatin) in 24-wells. Of the 6 clones, only one resulted in the correct sequence throughout the humanized region, which included the splice sites, and 188 bp immediately upstream, and another 182 bp immediately downstream of the humanized DNA: clone mEMS6759.

Clone mEMS6759 was further expanded and microinjected into B6 albino blastocysts, which were subsequently implanted into pseudo pregnant females. A total of two chimeras, scored by coat colour, were produced, bred on the B6 background, and one chimera produced germline N1 pups. The new strain is B6-*Pax6* ^*FHe9-*^ (officially C57BL/6J-*Pax6* ^em8(PAX6*R240X)Ems^, RRID:MMRRC_071760-JAX). N1 pups were genotyped to reconfirm proper insertion of the humanization event (Figure 1B), and were sequenced (350 bp on each side of the variant, which included the splice sites) to reconfirm no undesired changes were present in the region surrounding the humanization (Figure 1C). The B6-*Pax6* ^*FHe9-*^ strain was then bred onto the 129 background to derive 129-*Pax6* ^*FHe9-*^ (officially 129-*Pax6* ^em8(PAX6*R240X)Ems^, RRID:MMRRC_071761-JAX). Both the B6 and 129-*Pax6* ^*FHe9-*^ strains are available from MMRRC.

### Cohorts 1 and 2 were homozygous for humanization and heterozygous for the FLAG tag and *Pax6* variant

To generate experimental cohorts of CHuMMMs, the newly developed 129-*Pax6* ^*FHe9−/+*^ strain was bred to the 129-*Pax6* ^*He9+/He9+*^ control strain, and subsequently to the B6-*Pax6* ^*He9+/He9+*^ control strain to produce the cohort: B6129F1-*Pax6* ^*He9+/He9+*^ controls and B6129F1-*Pax6* ^*FHe9-/He9+*^ experimentals (Figure 2A). Breeding and experimental mice were confirmed by genotyping using a combination of the inside-human assay to confirm the humanization event (Figure 2B), and *Ava*I/*Dde*I RFLP assay to distinguish between variant and non-variant alleles (Figure 2C). Controls were homozygous for the humanized *Pax6* exon 9, non-variant, while experimentals were homozygous for the humanized *Pax6* exon 9, but heterozygous for the linked FLAG tag and patient variant. Note: expression of the FLAG tag would be prevented by the presence of the patient variant. The new *Pax6* ^*FHe9-*^ allele was produced and expanded on the B6 background, and backcrossed and expanded on the 129 background, so it could be studied on a wide variety of genetic backgrounds. Here we chose to characterize the Pax6^*FHe9-*^ allele on the B6129F1 defined hybrid background.

**FIGURE 2:**
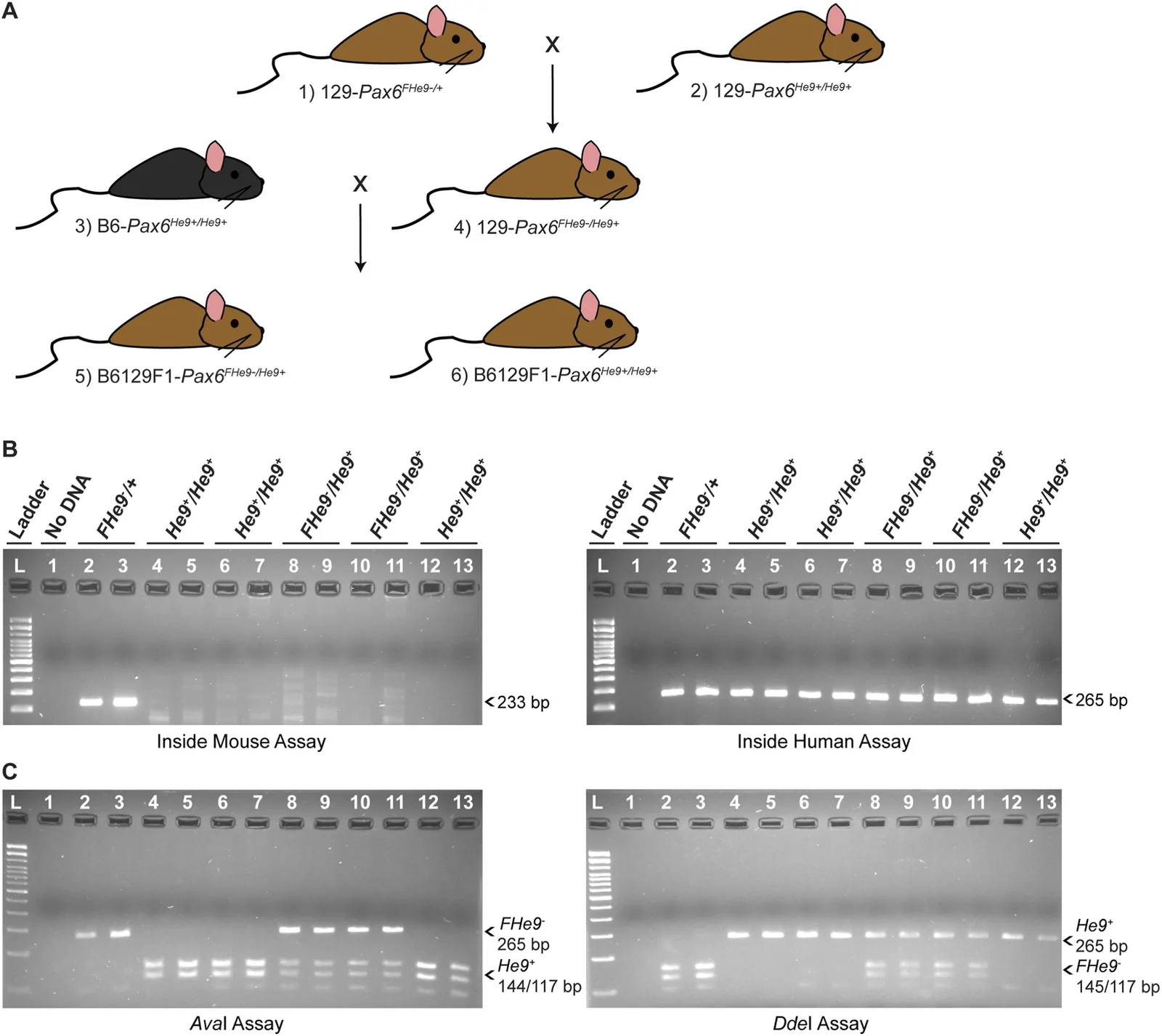
Breeding strategy for the new mouse model of aniridia. **(A)** Schematic of the experimental breeding strategy. The *Pax6* ^*FHe9-*^ allele was generated and maintained on the 129-agouti background (brown). Heterozygous 129-*Pax6* ^*FHe9−/+*^ female mice were mated with homozygous 129-*Pax6* ^*He9+/He9+*^ male mice to generate 129-*Pax6* ^*FHe9-/He9+*^ offspring. Then the 129-*Pax6* ^*FHe9-/He9+*^ male mice were crossed to B6-*Pax6* ^*He9+/He9+*^ female mice (black) to produce the first generation of hybrid agouti B6129F1-*Pax6* ^*FHe9-/He9+*^ and B6129F1-*Pax6* ^*He9+/He9+*^ mice (brown). **(B,C)** Results of genotyping assays used for identifying the mice carrying the *FHe9* ^*-*^ and *He9* ^*+*^ alleles. The expected band sizes are noted on the right-hand side of each gel. Unlabelled bands are primer dimer. Lane L: 100 bp DNA ladder, 1: no DNA negative control. Each lane shows results from an individual mouse, lanes 2 and 3: 129-*Pax6* ^*FHe9−/+*^, 4 and 5: 129-*Pax6* ^*He9+/He9+*^, 6 and 7: B6-*Pax6* ^*He9+/He9+*^, 8 and 9: 129-*Pax6* ^*FHe9-/He9+*^, 10 and 11: B6129F1-*Pax6* ^*FHe9-/He9+*^, 12 and 13: B6129F1-*Pax6* ^*He9+/He9+*^. **(B)** Human-specific “Inside Mouse Assay” and mouse-specific “Inside Human Assay” shown in Figure 1A. **(C)** Restriction fragment length polymorphism assays to detect the presence or absence of the pathogenic patient variant.

### Visual scoring and coronal histology revealed phenotypes in all mutant eyes

Congenital aniridia is typically caused by dominant loss-of-function variants that lead to *PAX6* haploinsufficiency and panocular phenotypes (Pedersen et al., 2020). It was important to show that the new B6129F1-*Pax6* ^*FHe9-/He9+*^ mouse, which carried the mouse equivalent of the human c.718C>T, p. R240X variant, showed the typical phenotypes of a mouse model of aniridia. Thus, a subset of the cohort 1 mice was observed at 3 weeks (P21–22) (Supplementary Table 3). As expected, all B6129F1-*Pax6* ^*He9+/He9+*^ eyes (n = 84) were scored as “normal” with 0% cloudiness. All B6129F1-*Pax6* ^*FHe9-/He9+*^ eyes (n = 80) were scored as abnormal. Phenotypes ranged from small (n = 61), closed (n = 18), or enlarged (likely glaucoma) (n = 1), and small eyes ranged from 20% to 100% cloudiness (average 73%). There was no observed difference in body weight between the two genotypes B6129F1-*Pax6* ^*He9+/He9+*^ and B6129F1-*Pax6* ^*FHe9-/He9+*^ (average 10.75 vs. 10.63, P = 0.671, two-tailed t-test).

Also at 3 weeks, eyes were harvested and analysis of coronal images revealed that, as expected, all 32 B6129F1-*Pax6* ^*He9+/He9+*^ control eyes had normal morphology, whereas various structural abnormalities were identified in the retina, lens, and cornea of B6129F1-*Pax6* ^*FHe9-/He9+*^ mice (Figure 3). Eyes were classified into four groups: mild, moderate, strong, and severe. The classification was based on the size and the severity of the structural abnormalities in the eye. Histological analysis of 68 B6129F1-*Pax6* ^*FHe9-/He9+*^ eyes showed that 18 had mild structural defects (e.g., small size, thin corneal epithelial layer), 25 had moderate defects (e.g., small, keratolenticular adhesions), 11 had strong defects (e.g., small, lens hypoplasia, retinal dysplasia), and 14 had severe defects (notably very small, overall malformed).

**FIGURE 3:**
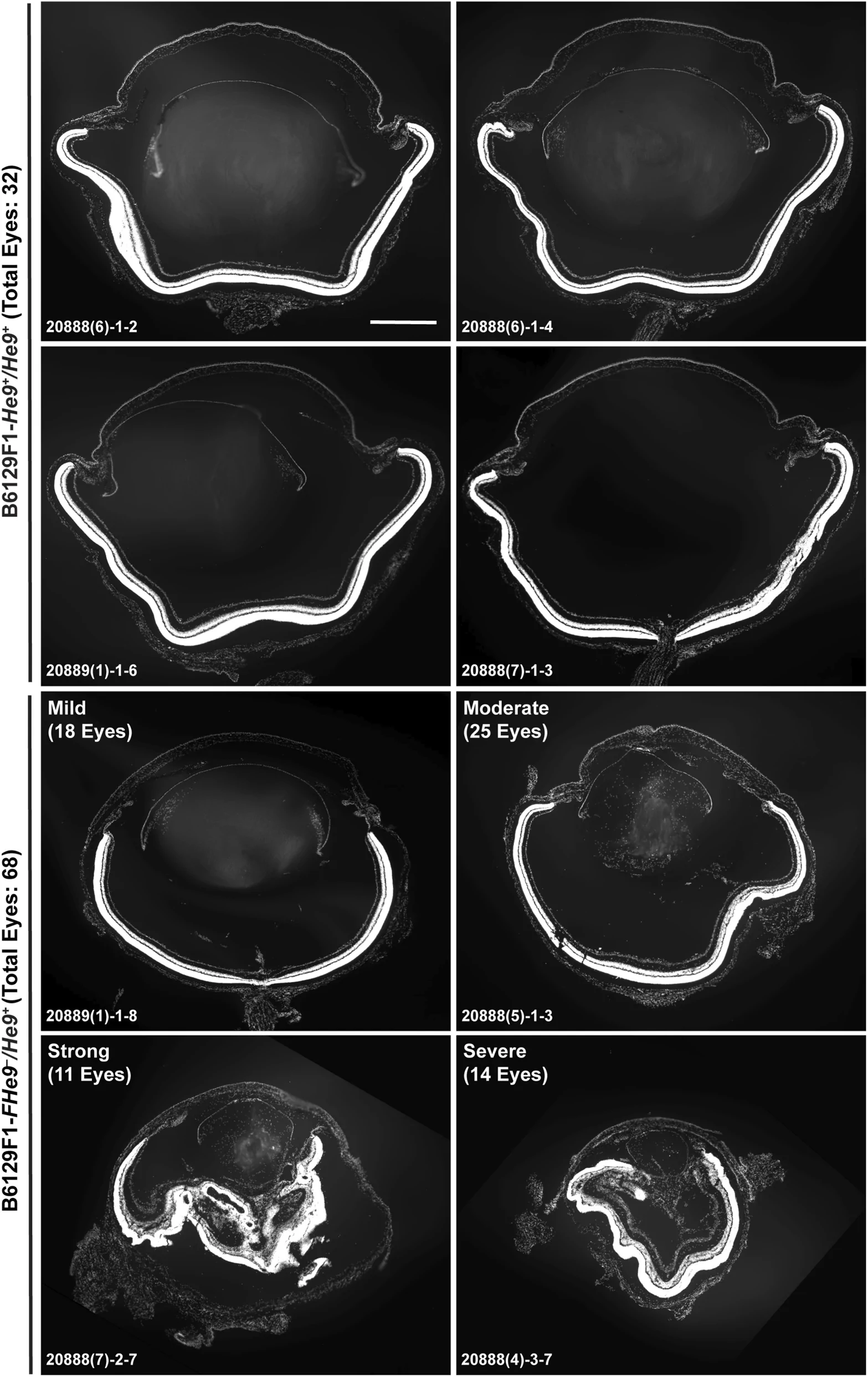
Fluorescent images revealed mutant eye phenotype ranging from mild to severe. Images of B6129F1-*He9* ^*+*^ */He9* ^*+*^ and B6129F1-*FHe9* ^*-*^ */He9* ^*+*^ mouse eyes at 21 days postnatal. Eyes were oriented to have the cornea on top and the retina on the bottom, and placed on a black background. Hoechst staining was used to visualize nuclei and overall tissue morphology. The mouse IDs are shown on the bottom of each image. The scale bar is 500 μm.

### Corneal abnormalities mimic those typically observed in patients

Keratopathy, including progressive corneal clouding and blood vessel incursion, is a major cause of blindness in aniridia and occurs in up to 90% of patients (Landsend et al., 2017). While visual inspection is useful for the initial evaluation of the eye in mice, it is less accurate than slit lamp imaging for examining the cornea. Therefore, we employed slit lamp imaging for longitudinal monitoring of the cornea in a subset of cohort 1 mice at different time points (3, 6, and 12 weeks) (Supplementary Table 3). As expected, all control B6129F1-*Pax6* ^*He9+/He9+*^ eyes exhibited clear corneas at every time point. In contrast, slit lamp imaging of B6129F1-*Pax6* ^*FHe9-/He9+*^ eyes revealed a high degree of keratopathy with corneal clouding and vascularization, which was present as early as 3 weeks and persisted through 12 weeks (Figure 4).

**FIGURE 4:**
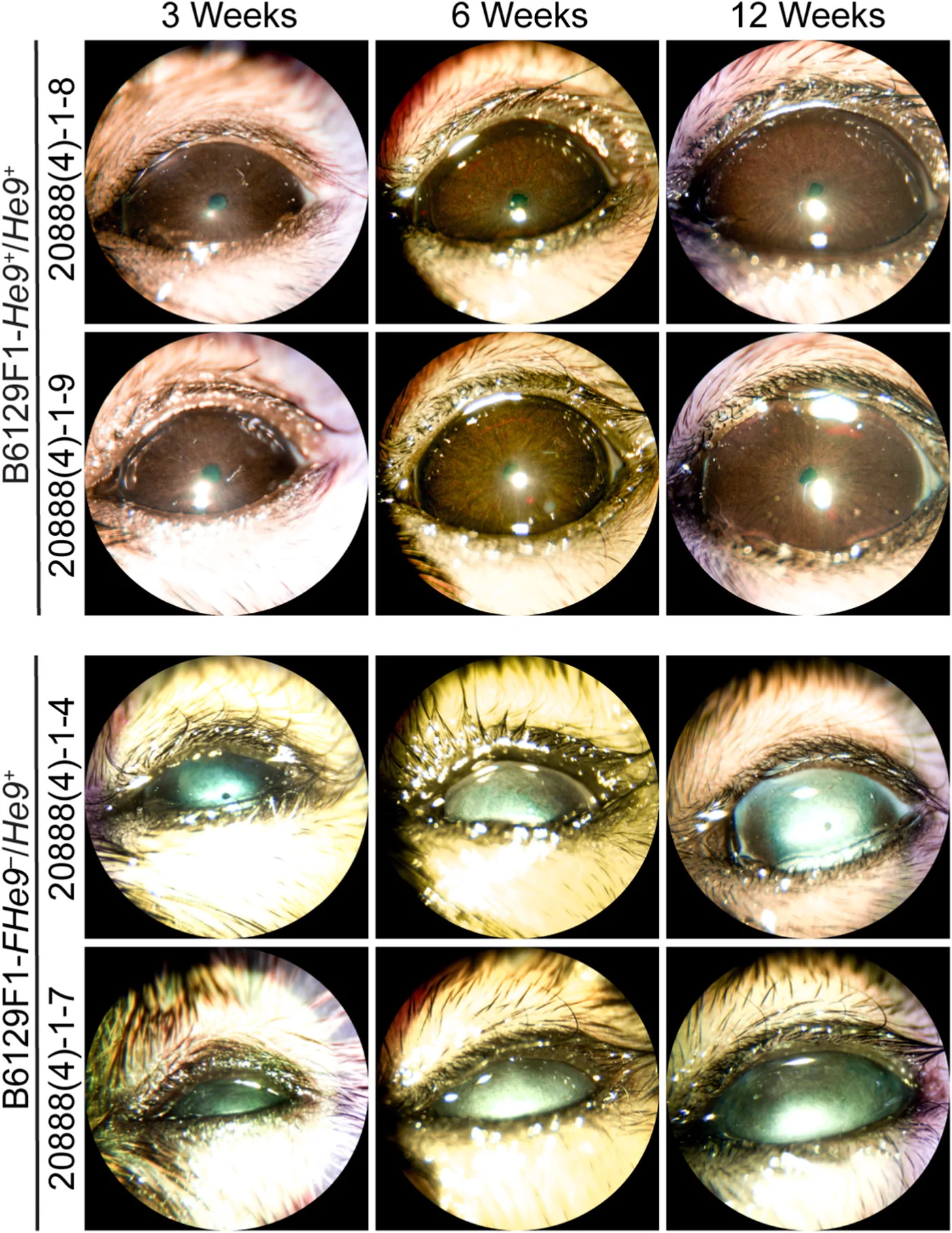
Slit-lamp images revealed anterior ocular phenotype present at 3 weeks and stable through 12 weeks. Sequential images of B6129F1-*He9* ^*+*^/*He9* ^*+*^ and B6129F1-*FHe9* ^*-*^/*He9* ^*+*^ mouse left eyes at 3, 6, and 12 weeks of age. For the *He9* ^*+*^ */He9* ^*+*^ control group, images are representative of 5 mice. For the *FHe9* ^*-*^/*He9* ^*+*^ group, images are representative of 4 mice. The mouse IDs are shown on the left side of each row.

### Minimal humanization does not affect *PAX6* expression or localization in the cornea and retina

Dominant loss-of-function variants that lead to *PAX6* haploinsufficiency would be expected to change the level of PAX6 protein, but not its expression pattern or localization. To check for this in the new humanized mouse model of aniridia, a subset of cohort 1 eyes was harvested at 12 weeks for confocal microscopy (Supplementary Table 3). The purpose was to examine whether minimal humanization of *Pax6* had caused misexpression or mislocalization of the protein. Corneal sections from both B6129F1-*Pax6* ^*He9+/He9+*^ and B6129F1-*Pax6* ^*FHe9-/He9+*^ mice showed the expected PAX6 expression in the corneal epithelium (Figure 5A). Furthermore, consistent with other mouse models of aniridia (Mirjalili Mohanna et al., 2022; Ramaesh et al., 2003), B6129F1-*Pax6* ^*FHe9-/He9+*^ mice showed thinner corneal epithelium compared to controls. Retinal sections from both groups showed the expected PAX6 expression in the ganglion cell layer and inner nuclear layer (Figure 5B). Our observations confirm that the minimal humanization of the *Pax6* gene does not interfere with the expression or localization of PAX6 protein in the cornea and the retina.

**FIGURE 5:**
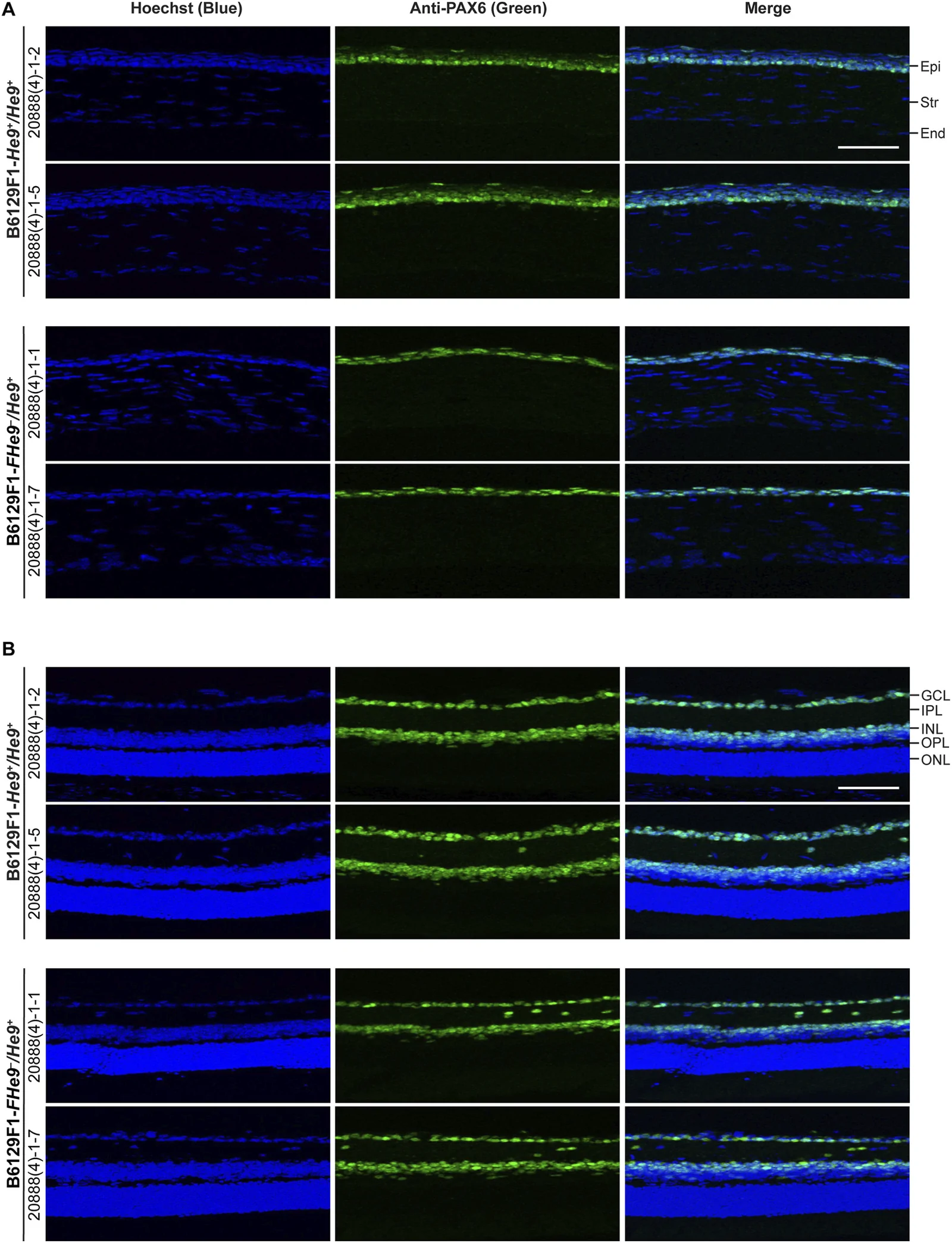
Confocal images reveal normal PAX6 expression and localization in humanized eyes. Images of B6129F1-*He9* ^*+*^ */He9* ^*+*^ and B6129F1-*FHe9* ^*-*^ */He9* ^*+*^ mouse **(A)** corneas and **(B)** retinas. For the *He9* ^*+*^/*He9* ^*+*^ control group, images are representative of 10 eyes. For the *FHe9* ^*-*^/*He9* ^*+*^ group, images are representative of 8 eyes. Corneal and retinal cells were identified by their location, morphology, and staining with anti-PAX6 antibody. All images were taken at the same magnification and light setting. The mouse IDs are shown on the left side of each row. The scale bar is 100 μm. End, endothelium; Epi, epithelium; GCL, ganglion cell layer; INL, inner nuclear layer; IPL, inner plexiform layer; ONL, outer nuclear layer; OPL, outer plexiform layer; Str, stroma.

### LNP delivery of ABE8e RNPs partially corrected the patient variant in *ex vivo* cortical neurons

*Ex vivo* experiments serve as a crucial first step toward *in vivo* studies, offering valuable insights into the efficacy of the CRISPR strategy and delivery method in a controlled environment before progressing to more complex *in vivo* models. Here we used a CRISPR strategy (Figure 6A), employing ABE8e, which in a previous study demonstrated superior editing of the *PAX6* c.718C > T variant in humanized mouse ESCs compared to *Sp*Cas9 (Adair et al., 2023). Moreover, we chose the Incisive Delivery System for our CRISPR-based genome editing, since it had been used successfully before to deliver ABE8e *ex vivo* to cortical neurons (Adair et al., 2023).

**FIGURE 6:**
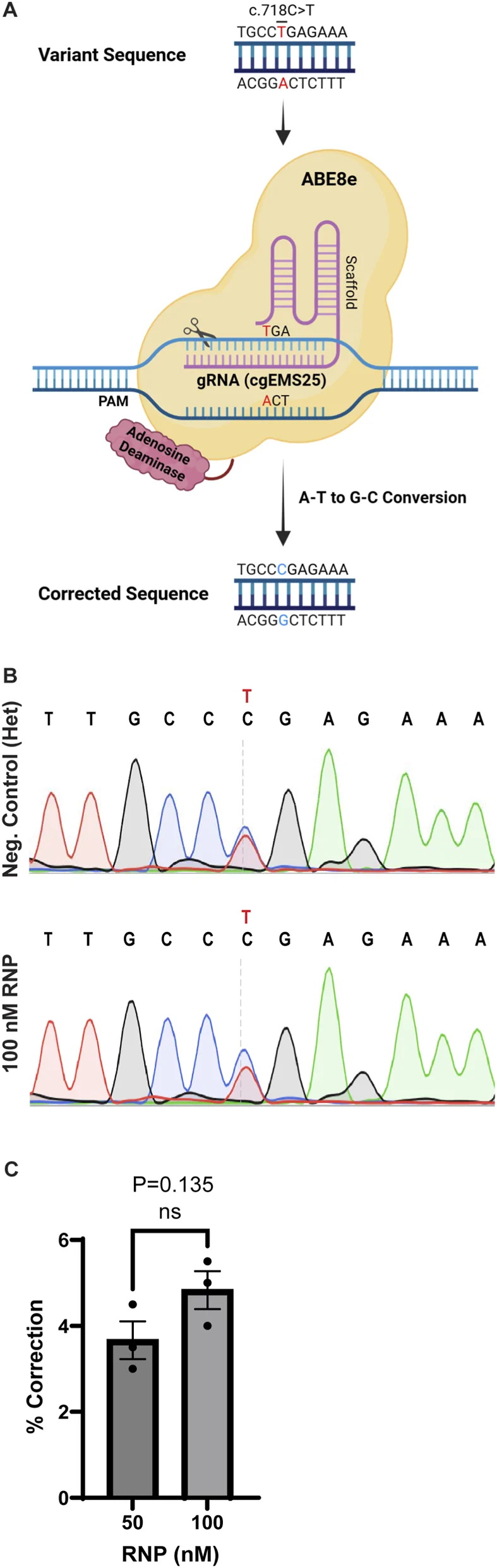
*Ex vivo* correction of the pathogenic *PAX6* variant in *FHe9* ^*-*^ */He9* ^*+*^ mouse cortical neurons using lipid nanoparticle (LNP)-encapsulated adenine base editor 8e (ABE8e) ribonucleoproteins (RNPs). **(A)** Schematic representation of the CRISPR base-editing strategy to correct the aniridia-associated *PAX6* variant (c.718 C>T, p. R240X). ABE8e and chemically modified guide RNA (gRNA) cgEMS25 are used to correct the variant from A:T to G:C base pairs without introducing double-stranded breaks or requiring donor DNA templates. Created using BioRender. M, Z. (2026) https://BioRender.com/r8ygh9y **(B)** Top panel shows a representative Sanger sequencing chromatogram encompassing the patient variant from *FHe9* ^*-*^ */He9* ^*+*^ cortical neurons treated with the luciferase non-targeting strategy (negative control). Bottom panel shows a representative chromatogram from *FHe9* ^*-*^ */He9* ^*+*^ cortical neurons treated with LNP-encapsulated ABE8e RNPs (100 nM). **(C)** Quantification of variant correction in *FHe9* ^*-*^ */He9* ^*+*^ cortical neurons treated with LNP-encapsulated ABE8e RNPs at either 50 nM or 100 nM. A two-tailed t-test was used to determine the significance of the data. Error bars indicate the standard error of the mean of three biological replicates. ns, non-significant.

LNP-delivered ABE8e RNPs were tested in two concentrations (50 nM and 100 nM) to examine the efficacy of this approach for variant correction at the DNA level. The *ex vivo* cortical neurons were isolated from B6129F1-*Pax6* ^*FHe9-/He9+*^ embryos and so were homozygous for the humanization of exon 9, but heterozygous at the pathogenic variant - carrying the non-variant C (blue) on one chromosome and the pathogenic variant T (red) on the other (Figure 6B). As expected, in the non-targeting luciferase negative control, the Sanger sequencing chromatogram shows the peaks are almost the same height. However, genome editing successfully increased the non-variant peak in the *Pax6* ^*FHe9-/He9+*^ *ex vivo* cortical neurons after a single treatment with 100 nM ABE8e RNPs (Figure 6B). No undesired base conversions were observed within the sequenced region. The sequence heterozygosity made it challenging to confidently interpret peak changes from some of the Sanger chromatograms, especially for the 50 nM condition. Thus, a blinded analysis was undertaken using EditR software for peak quantification, which was normalized against the luciferase targeting negative control, to determine the percentage of variant correction in the experimental samples (Supplementary Table 7). Sanger sequencing results from three biological replicates demonstrated successful variant correction. Higher average variant correction was observed for the 100 nM treatment (4.8% ± 0.4% SEM) compared to the 50 nM treatment (3.7% ± 0.4% SEM) (Figure 6C). This difference between groups was not statistically significant.

### Humanization does not significantly impact *Pax6*, but the FLAG tag does significantly

The question arose whether either of the components of the new allele, i.e. humanization or FLAG tag, impacted allele function? It had previously been demonstrated that *Pax6* ^*He9+*^, in which the humanization was added to a non-variant *Pax6*, resulted in no phenotype when carried heterozygously or homozygously (Adair et al., 2023). Similarly, it had previously been demonstrated that *Pax6* ^*Fax*^, in which the FLAG tag was added to a non-variant *Pax6*, resulted in no phenotype when carried heterozygously or homozygously (Mirjalili Mohanna et al., 2020; Mirjalili Mohanna et al., 2022). However, an even more rigorous test would be to breed mice in which only one allele is producing PAX6 protein. Thereby asking whether a Wt allele could be replaced with a modified allele, and if the modified allele would result in a more rapid or extreme phenotype at wean, remembering that *Pax6*-null is perinatal lethal with no eyes (Hickmott et al., 2018; Hil et al., 1991; Hogan et al., 1986). We addressed this question with two breeding experiments (Figure 7).

**FIGURE 7:**
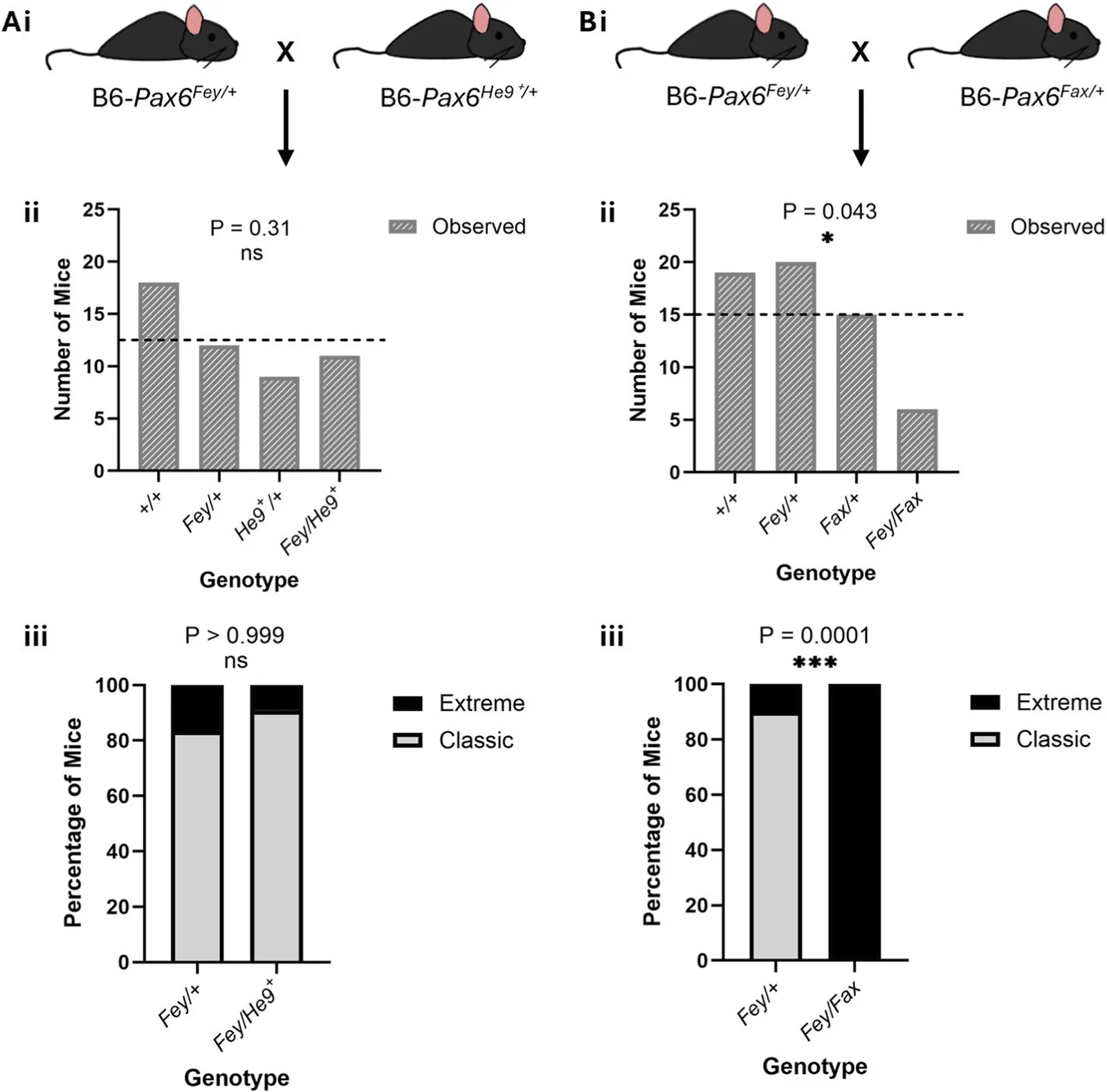
Breeding experiments to test the function of the humanized **(A)**, or FLAG-tagged *Pax6* **(B)** alleles. (Ai) Schematic of the breeding strategy to test humanization of *Pax6*. B6-*Pax6* ^*Fey/+*^ mice were crossed with B6-*Pax6* ^*He9+/+*^ mice and 50 pups were produced. (Aii) Numbers and genotypes: 18 *+*/*+*, 12 *Fey*/*+*, 9 *He9* ^*+/+*^, and 11 *Fey*/*He9* ^*+*^. Chi-square analysis showed no significant deviation from the expected 12.5 per genotype (χ^2^ = 3.6, df = 3, P = 0.31). (Aiii) Genotypes and phenotypes: *Fey*/*+*, 10 classic and 2 extreme; *Fey*/*He9* ^*+*^, 10 classic and 1 extreme. Two-tailed Fisher’s exact test showed no significant association between genotype and phenotype (P > 0.999). (Bi) Schematic of the breeding strategy to test the FLAG-tagged *Pax6*. B6-*Pax6* ^*Fey/+*^ mice were crossed with B6-*Pax6* ^*Fax/+*^ mice and 60 pups were produced. (Bii) Numbers and genotypes: 19 *+*/*+*, 20 *Fey*/*+*, 15 *Fax*/*+*, and 6 *Fey*/*Fax*. Chi-square analysis showed a significant deviation from the expected 15 per genotype (χ^2^ = 8.14, df = 3, P = 0.043). (Biii) Genotypes and phenotypes: *Fey*/*+*, 18 classic and 2 extreme; *Fey*/*Fax*, 6 extreme. Two-tailed Fisher’s exact test showed a highly significant association between genotype and phenotype (P = 0.0001). ns, non-significant.

First, we assessed the impact of humanization on *Pax6* allele function by examining mice generated from crosses between B6-*Pax6* ^*Fey*/+^ and B6-*Pax6* ^*He9+/+*^ (Figure 7Ai). To assess whether genotype frequencies deviated from Mendelian inheritance, observed genotypes from 50 mice were compared to the expected 1:1:1:1 ratio using a chi-square test (Figure 7Aii). The observed numbers were 18 *+*/*+*, 12 *Fey*/*+*, 9 *He9* ^*+/+*^, and 11 *Fey*/*He9* ^*+*^ mice. No significant deviation from Mendelian inheritance was detected, indicating that none of the genotypes were underrepresented. We then compared the ocular phenotype between *Pax6* ^*Fey*/+^ and *Pax6* ^*Fey/He9+*^ mice, since the *Fey* allele is known to produce no functional PAX6 protein (Figure 7Aiii). (Mirjalili Mohanna et al., 2020; Mirjalili Mohanna et al., 2022; Adair et al., 2023) Thus, we are comparing the function of Wt and *He9+* alleles alone. Mice were categorized based on their ocular phenotype. Pups exhibiting a small eye with or without corneal clouding, or unilateral closed eye, were categorized as having classic aniridia, whereas pups with bilateral closed eyes were categorized as having extreme aniridia. Among *Fey*/*+* mice (n = 12), 10 exhibited classic aniridia and 2 exhibited extreme aniridia. Among *Fey*/*He9* ^*+*^ mice (n = 11), 10 exhibited classic aniridia and 1 exhibited extreme aniridia. Two-tailed Fisher’s exact test revealed no significant association between genotype and aniridia severity category. Thus, the *Pax6* ^*He9+*^ produces PAX6 protein that is functionally indistinguishable from a Wt mouse allele, demonstrating there is no negative impact of the humanization on allele function.

Second, we assessed the impact of the FLAG tag on *Pax6* allele function by examining mice generated from crosses between B6-*Pax6* ^*Fey*/+^ and B6-*Pax6* ^*Fax/+*^ animals (Figure 7Bi). The *Pax6* ^*Fax*^ was the allele that the new *Pax6* ^*FHe9-*^ was built upon. To assess whether genotype frequencies deviated from Mendelian inheritance, observed genotypes from 60 mice were compared to the expected 1:1:1:1 ratio using a chi-square test (Figure 7Bii). The observed numbers were 19 *+*/*+*, 20 *Fey*/*+*, 15 *Fax*/*+*, and 6 *Fey*/*Fax* mice. A significant deviation from Mendelian inheritance was detected, suggesting an underrepresentation of mice with the *Fey/Fax* genotype. We next compared ocular phenotype between *Pax6* ^*Fey*/+^ and *Pax6* ^*Fey/Fax*^ mice (Figure 7Biii). Thus, we are comparing the function of Wt and *Fax* alleles alone. Among *Pax6* ^*Fey*/+^ mice (n = 20), 18 exhibited classic aniridia and 2 exhibited extreme aniridia, but all *Pax6* ^*Fey/Fax*^ mice (n = 6) exhibited extreme aniridia. Two-tailed Fisher’s exact test revealed a highly significant association between genotype and aniridia severity category. Thus, although *Pax6* ^*Fax*^ produces functional PAX6 protein, the allele is not able to fully replace a Wt mouse allele, demonstrating there is some negative impact of the FLAG tag on allele function.

## Discussion

We have developed a CHuMMM for aniridia; specifically, a mouse model designed for the development of genome editing therapies. This mouse has several novel features. First is the humanized single-exon “landing pad”, which allows genome editing therapies such as CRISPR, that depend upon a local PAM sequence and binding genomic DNA, to be developed on human DNA sequence. This makes therapies developed using these models immediately translatable to human cells or patients without the need for redesigning any components. Second, the *He9-* variant introduced into exon 9 has never been available in mice before, but is the most common recurring pathogenic aniridia-patient variant and thus an important target for therapy. Third, the variant allele carries a tightly-linked FLAG tag that becomes expressed upon correction. This design allows for the precise discrimination and quantification of the non-variant versus corrected variant PAX6 proteins, providing an accurate readout of therapeutic restoration in a setting that models patients who retain one functional allele. Finally, the CHuMMM provides all the usual advantages for therapy development offered by a mouse model, including the ability to test efficacy, safety, and *in vivo* delivery.

Although designed for the development of genome-editing therapies, the CHuMMMs humanization is equally important for other therapies that require an interaction with human DNA or RNA. For example, oligonucleotides to change splicing, antisense oligonucleotides to bind mRNA and inhibit translation, or enzyme-independent homologous recombination (Hiramoto et al., 2018). This mouse model will also be a new tool for testing nonsense suppression drugs, since the “read through” protein would be FLAG-tagged. Of course, these mice remain suitable for development of other non-DNA/RNA binding therapeutics, as with any typical mouse model. For example, augmentation gene therapy, surgical intervention, and small molecules.

The new *Pax6* ^*FHe9-*^ allele can be used in two chromosomal arrangements. First, mice can be bred to carry only the new *FHe9-* with a Wt mouse allele on the other chromosome (*Pax6* ^*FHe9−*/+^). This arrangement will be suitable when comparing the efficacy of different CRISPR strategies on human DNA. Second, as done here, mice can be bred to carry the new *FHe9-* allele on one chromosome, and the control *He9+* allele on the other chromosome (*Pax6* ^*FHe9-*/*He9+*^). These mice will be homozygous for humanization and heterozygous for the variant, paralleling patients. This arrangement will be suitable when comparing the efficacy of different CRISPR strategies, but also their ability to distinguish between the variant and non-variant alleles, a critical therapeutic requirement for dominant diseases such as aniridia (Adair et al., 2023; Gibson et al., 2025).

The new *Pax6* ^*FHe9-*^ allele was not expected, nor desired, to generate any new phenotypes, and it did not. Ocular assessment by gross examination, slit lamp imaging, and fluorescent and confocal microscopy revealed the expected phenotypes for a mouse model of aniridia (Mirjalili Mohanna et al., 2020; Javidjam et al., 2024; Ton et al., 1992). This included the expected variability seen in mouse models of aniridia (Hickmott et al., 2018). However, the impression of the phenotype of the *Pax6* ^*FHe9-*/*He9+*^ mouse was more rapid and severe than expected. This is best seen in Figures 3, 4, where at P21 strong and severe ocular phenotypes are observed. This is an uncertain observation, since a head-to-head comparison of the *Pax6* ^*FHe9-*/He9+^ mouse, with, for example the *Pax* ^*Sey*/*He9+*^ mouse, was not conducted. Still, this potential observation bears consideration, since for some experiments a rapid obvious development of phenotype is advantageous, but for other experiments a more human-like slower time course with a longer therapeutic window is advantageous. We will consider three hypotheses to explain this potential rapid and severe phenotype: 1) incomplete backcross, 2) a more severe variant, 3) multiple modified *Pax6* alleles.

Incomplete backcrossing is a strong hypothesis to explain the potential observation of rapid and severe phenotypes. It is well known that genetic background affects the speed and severity of phenotype in the *Sey* aniridia mouse model (Hickmott et al., 2018). In general terms, the B6 background introduces considerable variability and severity of microphthalmia and structural perturbations. In comparison, the B6129F1 defined hybrid and 129 backgrounds slow and normalize the phenotype. Here we expected results similar to the B6129F1 background, but in fact observed results similar to the B6 background. This can be attributed to our incomplete backcrossing where, instead of invariant 50% B6 and 50% 129, on average the mice were 68.75% B6 and 31.25% 129. Given this is an average, some mice would have carried even more B6 genes, and thus would be expected to display the B6 severe phenotype. We conclude that the *FHe9-* allele responds to the genetic background similarly to the *Sey* allele. Thus, for future experiments, if a more rapid and severe phenotype is desired, the B6 background is appropriate, but if a more human-like slower time course is desired, a fully backcrossed B6129F1 or 129 background is optimal. The strains are currently being further backcrossed at the MMRRC to ensure genetic consistency and reduced variability for future studies.

Could the *FHe9-* allele itself be more severe than *Sey*? We find no support for this hypothesis. Both alleles are a single base-pair change that results in a premature stop codon; *Sey* is in exon 8, and *He9-* is in exon 9. Thus, they are only 45 amino acids apart in the protein, which is a total 422 (classical) or 436 (5a) amino acids. Could abnormal splicing due to humanization make *FHe9-* more severe? Using exactly the same humanization methodology, we generated *He9+* without the pathogenic variant (Adair et al., 2023). We previously characterized that allele extensively (Adair et al., 2023) and, in this work, demonstrated its completely normal functionality. We further confirmed the identity of the *He9+* and *FHe9-*humanizations by sequencing, including the conserved splice sites, and therefore have no basis to expect a difference in splicing. Although there is no support for a truncated *PAX6* protein exerting a dominant-negative effect in *Sey* (Mirjalili Mohanna et al., 2020; Hickmott et al., 2018), if one does exist, the *FHe9-* fragment would be quite similar. Since both alleles have equivalents in the human genome, we can also compare the patients. In the same paper, there is a comparison of one patient with the *Sey* equivalent variant (c.580G>T, p. G194X) and 6 patients with the *He9-* equivalent variant (c.718C>T, p. R240X). Although the phenotypes are variable, they do not provide evidence that the *He9-* is more severe (Kit et al., 2021).

We undertook breeding experiments to address the impact of multiple modified *Pax6* alleles (Figure 7), and whether that could be responsible for the potential observation of rapid and severe phenotypes. We did not find support for this hypothesis.

Given the complete conservation of amino acids, and high level of DNA conservation in the splice site sequences, we did not expect, nor did we see, an impact of the humanization alone. Previous work had shown that the *Pax6* ^*He9+*^ allele resulted in no phenotype when carried heterozygously or homozygously (Adair et al., 2023). Here we tested the Wt mouse allele compared to the *Pax6* ^*He9+*^ allele by having them on one chromosome and *Fey*, a non-functional allele, on the other: *Pax6* ^*Fey*/+^ and *Pax6* ^*Fey/He9+*^. In the resulting mice, we saw the expected representation of genotype and the classic aniridia-like phenotype, with no increase in severity as would be expected if the *Pax6* ^*He9+*^ allele had reduced function.

The same breeding experiment was conducted for *Pax6* ^*Fax*^, which similarly had previous work showing no phenotype when carried heterozygously or homozygously (Mirjalili Mohanna et al., 2020; Mirjalili Mohanna et al., 2022). Here we tested the Wt mouse allele compared to the *Pax6* ^*Fax*^ allele by again having them on one chromosome and *Fey* on the other: *Pax6* ^*Fey*/+^ and *Pax6* ^*Fey/Fax*^. In the resulting mice, we saw a significant deviation from the expected representation of genotype, suggesting an underrepresentation of mice with the *Pax6* ^*Fey/Fax*^ genotype. Since genotyping was at wean, we assume these pups died pre-wean. In addition, we observed a significant increase in phenotypic severity with all the *Pax6* ^*Fey/Fax*^ mice exhibiting extreme aniridia. Thus, although the *Pax6* ^*Fax*^ allele produces a functional protein, the allele has reduced function. This result acts as a positive control for the humanization breeding experiment, showing that allele modification can reduce function, but critically, it does not explain the potentially more rapid and severe phenotype in the *Pax6* ^*FHe9-*/He9+^ mice. This is because the FLAG-tagged *Pax6* would not be expressed due to the presence of the premature stop-codon pathogenic patient variant.

These breeding experiment results raise the question of whether genome editing correction of the *Pax6* ^*FHe9-*^ allele has the potential to fully correct the aniridia-like phenotype. Genome correction would change the *Pax6* ^*FHe9-*^ non-expressing allele, into a *Pax6* ^*FHe9+*^ expressing allele. Given that *Pax6* ^*Fax*^ is not equivalent to a Wt mouse allele, we must conclude that *Pax6* ^*FHe9+*^ would also not be equivalent to Wt. However, the CHuMMMs allele configuration is either *Pax6* ^*FHe9−*/+^ or *Pax6* ^*FHe9-*/He9+^, thus the corrected combination would be either *Pax6* ^*FHe9+/+*^ or *Pax6* ^*FHe9+*/He9+^. As demonstrated above, the concern is not based on the presence of the humanization but rather the FLAG tag. So would *Pax6* ^*Fax/+*^ or *Pax6* ^*Fax/He9+*^ have a phenotype. For *Pax6* ^*Fax/+*^, previous work showed this heterozygote had no phenotype (Mirjalili Mohanna et al., 2020; Mirjalili Mohanna et al., 2022). For *Pax6* ^*Fax/He9+*^ the breeding experiment has not been done, but we expect no phenotype because we have shown here that the *Pax6* ^*He9+*^ allele is equivalent to a Wt mouse allele. Thus, we conclude that correction of the variant would indeed have the potential to fully rescue the phenotype back to Wt.

Here we demonstrate the utility of the CHuMMM by deriving clinically relevant *ex vivo* cortical neurons and testing a CRISPR strategy using LNP-delivered ABE8e. This approach achieved modest variant correction (4.8% ± 0.4% SEM), which was not further optimized in a target cell type. Analysis of the region surrounding the variant in *ex vivo* cortical neurons revealed no undesired base conversions, including potential bystander edits. These results differ from our previous work using closely related alleles and ABE8e, where higher editing efficiencies were achieved but bystander edits were observed in mouse ESCs, and where re-expression of the protein was demonstrated by FLAG tag detection in cortical neurons (Adair et al., 2023). A limitation of the current study is that we examined only the DNA editing frequency, but this time did not demonstrate expression of the corrected protein by FLAG tag detection. Also, future studies would benefit from employing site-specific next-generation sequencing for more sensitive detection of genomic manipulation outcomes (Mirjalili Mohanna et al., 2020).

If one considers a cellular therapy in which *ex vivo* gene editing is performed, for example, in limbal stem cells before they are returned to the patient (Naderi and Ahmad, 2026), then a correction rate of 4.8% would be suitable for the isolation of completely corrected clones. However, cellular therapy has not yet been established for aniridia (Naderi and Ahmad, 2026; van Velthoven et al., 2023; Landsend et al., 2021). In considering *in vivo* gene therapy, first parameter-optimization experiments would be needed, including determining the efficiency in different target cells, since correction may vary between cell types (Mandal et al., 2014). Additionally, it will be important to explore various delivery vehicles (e.g. LNP versus rAAV) and delivery routes (e.g. intravenous versus intrastromal). The exact level of PAX6 restoration required for *in vivo* therapeutic benefit remains unknown. Two recent *PAX6* augmentation gene-therapy studies in a haploinsufficient mouse model of aniridia demonstrated that an approximately 7%–9% increase in PAX6 transcription was associated with measurable improvements in ocular phenotype at both the structural and molecular levels (Kaad et al., 2026; Djaksigulova et al., 2026). This suggests that complete restoration of PAX6 dosage may not be required for biological benefit. However, these findings cannot be directly compared with the genome editing efficiency determined here, and therefore the relationship between corrected allele frequency, restored PAX6 expression, and phenotypic rescue remains to be established.

Off-target analysis was not performed in the present study, as the primary focus was on mouse model development, with the variant correction experiment included as an exploratory proof-of-principle rather than an optimization effort. Importantly, the CHuMMM contains a minimally humanized region within an otherwise mouse genome. Therefore, once optimized CRISPR strategies have been developed, comprehensive off-target analyses will be necessary in relevant human cells to ensure specificity and safety.

Together, these data provide sufficient evidence that this strain represents a robust and appropriate model of the disease. While the phenotypic analyses and experimental breeding performed here establish the validity of this model, we acknowledge that further detailed functional characterization (e.g. electroretinography, transepithelial electrical resistance measurements, and optokinetic tracking) will be required, particularly in the context of future preclinical studies, to fully evaluate therapeutic outcomes.

## Conclusion

The aim of this study was to generate a new mouse model of aniridia: a CHuMMM. This mouse carries a minimally humanized FLAG-tagged *Pax6*, with the most common recurring pathogenic variant for aniridia. The mouse was designed and tested for the preclinical development of CRISPR-based gene therapies. During development, such therapies would bind human DNA in the mouse and thus be readily translatable to human cells and patients. Importantly, this mouse model will also be useful for other DNA and RNA binding therapeutic strategies. In addition, it is a new tool for testing nonsense suppression drugs. Finally, this mouse model remains suitable for development of other non-DNA/RNA binding therapeutics, as with any typical mouse model. Considering the broader impact of this work, it lays the foundation for the CHuMMMs approach for other pathogenic variants for aniridia in the same exon by CRISPR editing the control strain. It also sets an example for humanizing variants in other *PAX6* exons, and the regulatory region for CRISPRa. Finally, the CHuMMMs approach is widely applicable to other genetic diseases for which CRISPR-based therapy holds promise.

## Data Availability

The original contributions presented in the study are included in the article/Supplementary Material, further inquiries can be directed to the corresponding author. All mice used in this study are available from the not-for-profit distributors: JAX, Bar Harbour, ME, United States; or the MMRRC, Davis, CA, United States.
