# Supplementary material for: A novel minimally humanized mouse model of aniridia for preclinical evaluation of CRISPR gene editing strategies
Source: Front Genome Ed. 2026 Sep 18;8:1910209. doi: 10.3389/fgeed.2026.1910209 (PMC13631079; doi:10.3389/fgeed.2026.1910209)
Supplement: Supplementary file 1 [file DataSheet1.pdf]

SUPPLEMENT FOR:

A Novel Minimally Humanized Mouse Model of Aniridia for Preclinical Evaluation of CRISPR Gene Editing Strategies

Seyedeh Zeinab Mirjalili Mohanna[a,b], Andrea J. Korecki[b], Pardis Kazemian[a,b], Tess C. Lengyell[b], Bethany A. Adair[a,b], Blair R. Leavitt[a,b,c,d], Elizabeth M. Simpson[a,b,*]

[a]Department of Medical Genetics, The University of British Columbia, Vancouver, V6T 1Z3, Canada,

[b]Centre for Molecular Medicine and Therapeutics at BC Children's Hospital, The University of British Columbia, Vancouver, BC, V5Z 4H4, Canada,

[c]The Djavad Mowafaghian Centre for Brain Health, The University of British Columbia, Vancouver, BC, V6T 1Z3, Canada,

[d]Division of Neurology, Department of Medicine, University of British Columbia Hospital, Vancouver, BC, V6T 2B5, Canada.

[*]Corresponding Author. E-mail address simpson@cmmt.ubc.ca

Supplementary Table 1.
Guide RNAs and DNA templates used for *in vitro* and *ex vivo* CRISPR genome editing

ID	Description	Sequence (5'-3')
**cgEMS9**	gRNA for humanization of *Pax6* exon 9.	ACACUGUCAAGCUGUCUGAU
**cgEMS18**	gRNA for humanization of *Pax6* exon 9.	CAGUGACUGGUAUACAGCCA
**cgEMS25**	gRNA for correction of *PAX6* patient variant c.718C>T, p.R240X with ABE8e.	CUUUCUCAGGCAAACACAUC
**Luciferase**	gRNA against non-endogenous target (firefly luciferase) with no match in mouse.	CUUCGAAAUGUCCGUUGGGU
**oEMS6346**	512 base single-stranded DNA template for humanization of *Pax6* exon 9. This sequence contains the *PAX6* patient variant c.718C>T, p.R240X (bolded).	CCTTCGATTAGAAAACCATACCTGGAA<br>ATGCACAGAACAGGTTAGCCCTATGCT<br>TGCTACCACTCGGCCACAAACAAAAAG<br>CCCTCCATCCCCACCCTTACCTTTTTA<br>TTATATTAGTCCTATAAATAAATAGTA<br>CTCTGTACAAGCACCTCTGTCTCTAGG<br>AAAGACAAATGGTATGAATCACAAAGT<br>GTGAAACTGCACAGTCTCTCGGTACCT<br>GTATTCTTGCTTCAGGTAGATCTATTT<br>TGGCTGCTAGTCTTTCTC**A**GGCAAACA<br>CATCTGGATAATGGGTTCTCTCAAACT<br>CTGAAAGAGTAAGTTGATTTTCCATAT<br>TGTGCCAGAACTACACAAAATATGTTG<br>ACCAAACTGTGCATCAAACTGGTTCCC<br>ACCTCCCCACTCCCATTACCTCCAACC<br>AATTCCCACAGTGTGCTGACTGTACTA<br>GCAAGAACTTTCCCACCAGGAGCAAGT<br>TTTCTTTGGAATGACATTTAGTGTTTG<br>TCCTTATCTGTGGCCTAAGACAGTCA

ABE8e, adenine base editor 8e; cgEMS#, CRISPR guide EMS#; gRNA, guide RNA; ID, identifier; oEMS#, oligonucleotide EMS#.

Supplementary Table 2.
Primers and restriction enzymes used for PCR, RFLP, and Sanger sequencing

ID	Description	Sequence (5'-3')
**oEMS6223**	Forward primer for PCR screening of humanized *Pax6* exon 9; Forward primer for Sanger sequencing of humanized exon 9.	TTGGTTGGAGGTAATGGGAG
**oEMS6224**	Reverse primer for PCR screening of humanized *Pax6* exon 9; Reverse primer for Sanger sequencing of humanized exon 9.	CAAGCACCTCTGTCTCTAGG
**oEMS6220**	Forward primer for PCR screening of 5′ end of humanized *Pax6* exon 9.	TCTTCTTTTCACACACCAGC
**oEMS6244**	Reverse primer for PCR screening of 5′ end of humanized *Pax6* exon 9.	CCCCACCCTTACCTTTTTATTA
**oEMS6345**	Forward primer for PCR screening of 3′ end of humanized *Pax6* exon 9.	ACCGAGAGACTGTGCAGTTT
**oEMS6221**	Reverse primer for PCR screening of 3′ end of humanized *Pax6* exon 9.	CCTGTCTTCTCTGGTTCCTC
**oEMS6421**	Forward primer for PCR screening of mouse-specific sequence inside humanized *Pax6* exon 9.	GCTGTCTGATTGGCTGGATGA
**oEMS6422**	Reverse primer for PCR screening of mouse-specific sequence inside humanized *Pax6* exon 9.	GCATCCCAGTGCATAAAAACCA
***Ava*I**	Restriction enzyme used in RFLP to screen for correction of patient variant c.718C>T, p.R240X, in humanized *Pax6* exon 9 clones. Enzyme cuts (|) in presence of corrected base to give 144 bp and 117 bp fragments.	C|YCGRG<br>GRGCY|C
***Dde*I**	Restriction enzyme used in RFLP to screen for correction of patient variant c.718C>T, p.R240X in humanized *Pax6* exon 9 clones. Enzyme cuts (|) in presence of mutant base to give 145 bp and 117 bp fragments.	C|TNAG<br>GANT|C

ID, identifier; oEMS#, oligonucleotide EMS#; RFLP, restriction fragment length polymorphism.

Supplementary Table 3.
Mouse IDs, genotypes, gross assessments, and experimental procedure for cohort 1 mice

Mouse ID	Genotype	LE Size	LE Approx. % Cloudiness	RE Size	RE Approx. % Cloudiness	Weight (g)	Procedure
20888(4)-1-1	$FHe9^{-}/He9^{+}$	small	0	small	0	9.19	slit lamp (Fig 4), 12 w harvest, confocal (Fig 5)
20888(4)-1-2	$He9^{+}/He9^{+}$	normal	0	normal	0	9.41	slit lamp (Fig 4), 12 w harvest, confocal (Fig 5)
20888(4)-1-3	$He9^{+}/He9^{+}$	normal	0	normal	0	11.42	slit lamp (Fig 4), 12 w harvest, confocal (Fig 5)
20888(4)-1-4	$FHe9^{-}/He9^{+}$	small	0	small	0	10.80	slit lamp (Fig 4), 12 w harvest, confocal (Fig 5)
20888(4)-1-5	$He9^{+}/He9^{+}$	normal	0	normal	0	10.77	slit lamp (Fig 4), 12 w harvest, confocal (Fig 5)
20888(4)-1-6	$FHe9^{-}/He9^{+}$	small	0	small	0	9.02	slit lamp (Fig 4), 12 w harvest, confocal (Fig 5)
20888(4)-1-7	$FHe9^{-}/He9^{+}$	small	0	small	0	9.45	slit lamp (Fig 4), 12 w harvest, confocal (Fig 5)
20888(4)-1-8	$He9^{+}/He9^{+}$	normal	0	normal	0	9.44	slit lamp (Fig 4), 12 w harvest, confocal (Fig 5)
20888(4)-1-9	$He9^{+}/He9^{+}$	normal	0	normal	0	11.96	slit lamp (Fig 4), 12 w harvest, confocal (Fig 5)
20888(4)-2-1	$He9^{+}/He9^{+}$	normal	0	normal	0	10.14	P21 harvest, fluorescent (Fig 3)
20888(4)-2-2	$He9^{+}/He9^{+}$	normal	0	normal	0	11.42	P21 harvest, fluorescent (Fig 3)
20888(4)-2-3	$He9^{+}/He9^{+}$	normal	0	normal	0	11.13	P21 harvest, fluorescent (Fig 3)
20888(4)-2-4	$FHe9^{-}/He9^{+}$	closed	0	small	0	10.86	P21 harvest, fluorescent (Fig 3)
20889(1)-1-1	$He9^{+}/He9^{+}$	normal	0	normal	0	9.32	P21 harvest, fluorescent (Fig 3)
20889(1)-1-2	$FHe9^{-}/He9^{+}$	closed	0	closed	0	8.42	P21 harvest, fluorescent (Fig 3)
20889(1)-1-3	$FHe9^{-}/He9^{+}$	closed	0	closed	0	8.06	P21 harvest, fluorescent (Fig 3)
20889(1)-1-4	$He9^{+}/He9^{+}$	normal	0	normal	0	8.89	P21 harvest, fluorescent (Fig 3)
20889(1)-1-5	$He9^{+}/He9^{+}$	normal	0	normal	0	8.34	P21 harvest, fluorescent (Fig 3)
20889(1)-1-6	$He9^{+}/He9^{+}$	normal	0	normal	0	8.43	P21 harvest, fluorescent (Fig 3)
20889(1)-1-7	$FHe9^{-}/He9^{+}$	small	80	closed	0	8.46	P21 harvest, fluorescent (Fig 3)
20889(1)-1-8	$FHe9^{-}/He9^{+}$	closed	0	closed	0	8.35	P21 harvest, fluorescent (Fig 3)
20888(5)-1-1	$He9^{+}/He9^{+}$	normal	0	normal	0	10.78	P21 harvest, fluorescent (Fig 3)
20888(5)-1-2	$He9^{+}/He9^{+}$	normal	0	normal	0	10.38	P21 harvest, fluorescent (Fig 3)
20888(5)-1-3	$FHe9^{-}/He9^{+}$	small	100	small	100	12.22	P21 harvest, fluorescent (Fig 3)
20888(5)-1-4	$FHe9^{-}/He9^{+}$	small	80	small	100	10.71	P21 harvest, fluorescent (Fig 3)
20888(5)-1-5	$FHe9^{-}/He9^{+}$	closed	0	small	60	9.32	P21 harvest, fluorescent (Fig 3)
20888(5)-1-6	$He9^{+}/He9^{+}$	normal	0	normal	0	11.57	P21 harvest, fluorescent (Fig 3)
20888(5)-1-7	$FHe9^{-}/He9^{+}$	closed	0	closed	0	10.89	P21 harvest, fluorescent (Fig 3)
20888(5)-1-8	$FHe9^{-}/He9^{+}$	small	90	small	60	9.10	P21 harvest, fluorescent (Fig 3)
20888(5)-2-1	$FHe9^{-}/He9^{+}$	small	100	small	100	10.77	P21 harvest, fluorescent (Fig 3)
20888(5)-2-2	$He9^{+}/He9^{+}$	normal	0	normal	0	10.35	P21 harvest, fluorescent (Fig 3)
20888(5)-2-3	$He9^{+}/He9^{+}$	normal	0	normal	0	11.09	P21 harvest, fluorescent (Fig 3)
20888(6)-1-1	$FHe9^{-}/He9^{+}$	closed	0	closed	0	12.31	P21 harvest, fluorescent (Fig 3)
20888(6)-1-2	$He9^{+}/He9^{+}$	normal	0	normal	0	13.40	P21 harvest, fluorescent (Fig 3)
20888(6)-1-3	$FHe9^{-}/He9^{+}$	small	0	closed	0	11.79	P21 harvest, fluorescent (Fig 3)
20888(6)-1-4	$He9^{+}/He9^{+}$	normal	0	normal	0	13.10	P21 harvest, fluorescent (Fig 3)
20888(7)-1-1	$He9^{+}/He9^{+}$	normal	0	normal	0	9.60	P22 harvest, fluorescent (Fig 3)
20888(7)-1-2	$FHe9^{-}/He9^{+}$	small	100	small	100	9.52	P22 harvest, fluorescent (Fig 3)
20888(7)-1-3	$He9^{+}/He9^{+}$	normal	0	normal	0	10.34	P22 harvest, fluorescent (Fig 3)
20888(7)-1-4	$He9^{+}/He9^{+}$	normal	0	normal	0	11.30	P22 harvest
20889(3)-1-1	$He9^{+}/He9^{+}$	normal	0	normal	0	12.00	P22 harvest
20889(3)-1-2	$He9^{+}/He9^{+}$	normal	0	normal	0	11.20	P22 harvest
20889(3)-1-3	$FHe9^{-}/He9^{+}$	small	70	small	100	11.70	P22 harvest, fluorescent (Fig 3)
20888(7)-2-1	$FHe9^{-}/He9^{+}$	small	80	small	100	10.10	P21 harvest, fluorescent (Fig 3)
20888(7)-2-2	$He9^{+}/He9^{+}$	normal	0	normal	0	9.50	P21 harvest
20888(7)-2-3	$He9^{+}/He9^{+}$	normal	0	normal	0	9.45	P21 harvest
20888(7)-2-4	$He9^{+}/He9^{+}$	normal	0	normal	0	9.50	P21 harvest
20888(7)-2-5	$He9^{+}/He9^{+}$	normal	0	normal	0	10.60	P21 harvest
20888(7)-2-6	$He9^{+}/He9^{+}$	normal	0	normal	0	9.10	P21 harvest
20888(7)-2-7	$FHe9^{-}/He9^{+}$	small	80	small	80	10.40	P21 harvest, fluorescent (Fig 3)
20888(7)-2-8	$FHe9^{-}/He9^{+}$	small	100	small	80	8.80	P21 harvest, fluorescent (Fig 3)
20889(4)-1-1	$He9^{+}/He9^{+}$	normal	0	normal	0	11.26	P21 harvest
20889(4)-1-2	$FHe9^{-}/He9^{+}$	small	80	small	100	11.23	P21 harvest, fluorescent (Fig 3)
20889(4)-1-3	$FHe9^{-}/He9^{+}$	small	50	small	80	10.85	P21 harvest, fluorescent (Fig 3)
20889(4)-1-4	$He9^{+}/He9^{+}$	normal	0	normal	0	12.35	P21 harvest
20889(4)-1-5	$He9^{+}/He9^{+}$	normal	0	normal	0	11.32	P21 harvest
20889(2)-1-1	$FHe9^{-}/He9^{+}$	small	40	small	80	11.09	P22 harvest, fluorescent (Fig 3)
20889(2)-1-2	$FHe9^{-}/He9^{+}$	small	20	small	40	12.92	P22 harvest
20889(2)-1-3	$FHe9^{-}/He9^{+}$	small	40	small	40	11.23	P22 harvest, fluorescent (Fig 3)

Mouse ID	Genotype	LE Size	LE Approx. % Cloudiness	RE Size	RE Approx. % Cloudiness	Weight (g)	Procedure
20889(2)-1-4	$He9^+/He9^+$	normal	0	normal	0	10.94	P22 harvest
20889(2)-1-5	$FHe9^-/He9^+$	small	80	closed	0	11.35	P22 harvest, fluorescent (Fig 3)
20889(2)-1-6	$He9^+/He9^+$	normal	0	normal	0	11.60	P22 harvest
20889(2)-1-7	$He9^+/He9^+$	normal	0	normal	0	11.32	P22 harvest
20889(2)-1-8	$He9^+/He9^+$	normal	0	normal	0	12.03	P22 harvest
20889(2)-1-9	$FHe9^-/He9^+$	small	40	glaucoma	NA	11.89	P22 harvest
20889(2)-1-10	$FHe9^-/He9^+$	small	40	small	40	12.23	P22 harvest, fluorescent (Fig 3)
20889(2)-1-11	$FHe9^-/He9^+$	small	40	small	60	10.56	P22 harvest, fluorescent (Fig 3)
20888(4)-3-1	$He9^+/He9^+$	normal	0	normal	0	11.45	P21 harvest
20888(4)-3-2	$He9^+/He9^+$	normal	0	normal	0	11.76	P21 harvest
20888(4)-3-3	$He9^+/He9^+$	normal	0	normal	0	10.92	P21 harvest
20888(4)-3-4	$He9^+/He9^+$	normal	0	normal	0	10.22	P21 harvest
20888(4)-3-5	$FHe9^-/He9^+$	closed	0	small	100	10.79	P21 harvest, fluorescent (Fig 3)
20888(4)-3-6	$FHe9^-/He9^+$	small	80	small	60	11.38	P21 harvest, fluorescent (Fig 3)
20888(4)-3-7	$FHe9^-/He9^+$	closed	0	small	100	12.60	P21 harvest, fluorescent (Fig 3)
20888(4)-4-1	$He9^+/He9^+$	normal	0	normal	0	11.24	P21 harvest
20888(4)-4-2	$He9^+/He9^+$	normal	0	normal	0	11.21	P21 harvest
20888(4)-4-3	$FHe9^-/He9^+$	small	80	small	60	11.81	P21 harvest, fluorescent (Fig 3)
20888(4)-4-4	$FHe9^-/He9^+$	small	80	small	100	8.76	P21 harvest, fluorescent (Fig 3)
20888(4)-4-5	$FHe9^-/He9^+$	small	100	small	60	10.85	P21 harvest, fluorescent (Fig 3)
20888(4)-4-6	$FHe9^-/He9^+$	small	80	small	80	12.43	P21 harvest, fluorescent (Fig 3)
20888(4)-4-7	$FHe9^-/He9^+$	closed	0	small	80	11.65	P21 harvest, fluorescent (Fig 3)
20888(4)-4-8	$FHe9^-/He9^+$	small	80	small	60	11.47	P21 harvest, fluorescent (Fig 3)

Approx., approximate; glaucoma, enlarged eye suggestive of glaucoma; ID, identifier; LE, left eye; NA, not applicable; P, postnatal day; RE, right eye; w, week.

Supplementary Table 4.
Particle size characterization of LNP-encapsulated ABE8e RNPs after transport and used in *ex vivo* experiment

Condition	Z-average (nm)	PDI	Dn 90 (nm)
**TCV Only**	53.4 ± 0.380	0.410 ± 0.0039	22.9 ± 1.42
**RNP - 50 nM**	1660 ± 125	0.553 ± 0.117	1070 ± 23.5
**RNP - 100 nM**	1880 ± 87.9	0.728 ± 0.0781	988 ± 96.2

Each sample was measured three times where the represented numbers are mean and standard error of the mean. ABE8e, adenine base editor 8e; Dn 90, estimated size of 90 percentile of particles; PDI, polydispersity index; RNP, ribonucleoprotein; TCV, transfection-competent vesicles.

Supplementary Table 5.
Particle size characterization of LNP-encapsulated ABE8e RNPs with no transport and not used in *ex vivo* experiment

Condition	Z-average (nm)	PDI	Dn 90 (nm)
**TCV Only**	53.5 ± 0.208	0.398 ± 0.0114	22.2 ± 7.96
**RNP - 50 nM**	317.1 ± 16.03	0.394 ± 0.076	74.97 ± 3.80
**RNP - 100 nM**	997.5 ± 63.57	0.719 ± 0.191	649.1 ± 25.13

Each sample was measured three times where the represented numbers are mean and standard error of the mean. ABE8e, adenine base editor 8e; Dn 90, estimated size of 90 percentile of particles; PDI, polydispersity index; RNP, ribonucleoprotein; TCV, transfection-competent vesicles.

Supplementary Table 6.
Summary of embryonic stem cell clones after CRISPR-mediated modification of *Pax6* exon 9

Clones Picked	224
Frozen & Harvested (96 Well)	187/224
Positive for Inside-Human Assay	18/187
Positive for 5′ and 3′ Assays	6/18
Frozen & Harvested (24 Well)	6/6
Sequencing Clean	1/6
Microinjected	1
Chimeras Produced	2
Chimeras That Went Germline	1

Supplementary Table 7.
Correction values for the *ex vivo* genome editing of *FHe9⁻/He9⁺* cortical neurons

Replica #	RNP Conc.	Forward Strand Cor. (%)	Reverse Strand Cor. (%)	Average Strand Cor. (%)	Average Replica Cor. (%) ± SEM
**1**		4.3	4.7	4.5	
**2**	50 nM	4.3	2.7	3.5	3.7 ± 0.4
**3**		4.3	1.7	3.0	
**1**		7.3	2.7	5.0	
**2**	100 nM	5.3	2.7	4.0	4.8 ± 0.4
**3**		5.3	5.7	5.5	

Cor., correction; RNP, ribonucleoprotein; SEM, standard error of the mean.
